# A Multimodal Workshop to Improve Medical Student Self-Assessment of Knowledge and Comfort Managing Patients With Suicidality

**DOI:** 10.15766/mep_2374-8265.11488

**Published:** 2025-01-17

**Authors:** Paige C. Chardavoyne, Amanda Liewen, Paige McKenzie, Jill Sorby, Ana Navarro, Julie R. Owen

**Affiliations:** 1 Fourth-Year Resident, Department of Psychiatry and Behavioral Medicine, Medical College of Wisconsin; 2 Assistant Professor, Department of Psychiatry and Behavioral Medicine, Medical College of Wisconsin; 3 Third-Year Resident, Department of Psychiatry and Behavioral Medicine, Medical College of Wisconsin; 4 Assistant Professor, Department of Psychiatry and Behavioral Medicine, Medical College of Wisconsin; Inpatient Psychiatrist, Clement J. Zablocki VA Medical Center; 5 Assistant Professor, Department of Psychiatry and Behavioral Medicine and Department of Emergency Medicine, Medical College of Wisconsin

**Keywords:** Suicide, Risk Assessment, Safety Planning, Psychiatry, Case-Based Learning, Self-Assessment, Standardized Patient

## Abstract

**Introduction:**

Suicide is a preventable public health concern, yet suicide remains a leading cause of death for Americans. Physicians are well positioned to screen for and address suicidality. It is important to provide formal suicide education during medical school so that physicians are better prepared to address this topic with their patients. The goal of this workshop was to improve medical student self-assessment of knowledge and comfort managing patients with suicidality.

**Methods:**

This was a 2.5-hour multimodal workshop developed for formal third-year medical student suicide education that included: flipped learning with a preworkshop presentation; experiential learning, scaffolding, reflection, and feedback with two standardized patient encounters; think-pair-share with small- and large-group discussion; and a facilitator-led large-group didactic.

**Results:**

Of the 105 third-year medical students who completed the workshop in the first half of the 2023–2024 academic year, 84% (88/105) completed the presurvey and 70% (73/105) completed the postsurvey. Prior to the workshop, on a 5-point Likert scale (1 = *not at all*, 5 = *extremely*), respondents rated their knowledge regarding suicide assessment as 2.2 and their comfort managing suicidality as 2.6. This improved to 4.0 and 3.9, respectively, on the postworkshop survey. Students felt the workshop was helpful and relevant.

**Discussion:**

Future work should involve identifying if this reported self-improvement transfers to clinical skill advancement and, if so, measuring the clinical impact of this innovation for patients. Furthermore, assessing if this self-reported improvement in knowledge and comfort managing suicidality is long-lasting should be explored.

## Educational Objectives

By the end of this activity, learners will be able to:

1. Obtain a history of present illness for a patient presenting with suicidal ideation.
2. Establish a leading diagnosis and differential diagnosis for a patient presenting with suicidal ideation.
3. Reflect on aspects of history taking with a patient presenting with suicidal ideation that went well and areas for future improvement.
4. Construct aspects of a safety plan for a patient presenting with suicidal ideation.
5. Develop a suicide risk assessment (including acute and chronic risk levels, risk factors, and protective factors) and risk mitigation strategies for a patient presenting with suicidal ideation.
6. Examine for changes in comfort managing a patient presenting with suicidal ideation.

## Introduction

Suicide is a preventable public health concern that impacts millions. Suicide attempts are one of the leading causes of death in the general population and a more common cause of death in younger populations.^1^ According to the U.S. Centers for Disease Control and Prevention, of adults in 2022, 13.2 million seriously contemplated, 3.8 million planned for, and 1.6 million attempted suicide.^1^ Individuals can present to any care setting with suicidal ideation, not just mental health settings. Thus, it is imperative that future physicians have basic knowledge and comfort assessing and managing patients with suicidality. The third-year psychiatry clerkship represents an opportunity to expose future physicians of varying specialties to suicide training.

According to an AAMC Curriculum Inventory report showing data regarding clerkship requirements from 2012–2023, between 96% and 100% of medical schools had a required psychiatry clerkship. Some institutions utilized a block rotation model while others used a longitudinal approach.^2^ The Association of Directors of Medical Student Education in Psychiatry developed a list of objectives for the medical student psychiatry clerkship.^3,4^ This included 23 topics, three of which (psychiatric emergencies, mood disorders, and child and adolescent psychiatry) have specific objectives involving suicide education. These include the following: The student will “identify the clinical and demographic factors associated with a statistically increased risk of suicide in general and clinical populations,” “develop a differential diagnosis, conduct a clinical assessment, and recommend management for a patient exhibiting suicidal thoughts or behavior,” “discuss the identification and management of suicide risk in general medical settings,” and “discuss the epidemiology and clinical features of suicide risk in adolescents.”^4^

Despite these objectives for medical students, research suggests that medical school psychiatry curricula may not be meeting these objectives. Finnegan and colleagues surveyed a sample of primary care providers and found that 37% reported feeling neutral or uncomfortable talking to patients about suicide, citing factors such as “lack of training,” “unsure what to do next,” and “unsure what to say,” amongst others.^5^ Despite the stakes for future physicians and their future patients, medical schools may be relying on varying, informal clinical experiences for suicide education.

To ensure that all third-year medical students at our institution received formal suicide education during their psychiatry clerkship, the authors developed a suicide risk assessment and safety planning workshop. Workshop scenarios were adapted to medical students from a preexisting, unpublished training for emergency medicine residents at our institution. While the authors were aware of preexisting curricula for suicide risk assessment, they chose these scenarios given familiarity and easy adaptability to medical students.

At the beginning of this workshop, the authors expected that third-year medical students may have varying amounts of clinical and personal experience with suicidality. Little research exists regarding medical student attitudes, knowledge, and confidence managing aspects of suicidality. No needs assessment of this topic area for third-year medical students was identified during a literature review. However, Chuop and colleagues surveyed second-year medical students pre- and postdidactic session and standardized patient (SP) encounter that focused on suicide education and prevention techniques.^6^ Their results suggested that there are gaps in medical student familiarity with suicide risk assessments and confidence with safety planning.^6^ Thus, the present workshop was developed with risk assessments and safety planning as focal points.

SP exercises to promote medical students’ psychiatric history taking skills have been published in *MedEdPORTAL*,^7,8^ though SPs in the cited exercises did not present with suicidal ideation. Team-based learning (TBL) activities involving the assessment and management of patients with suicidal thoughts have been published,^9^ but subjective or objective improvements in suicide management were not quantitatively assessed. Studies support that medical students prefer multimodal approaches, consistent with the notion that students learn differently.^10^ With this in mind, the authors incorporated different educational techniques into the present workshop, including flipped classroom, didactics, SP encounters, and group discussions.

To the authors’ knowledge, this work is a novel, multimodal workshop for third-year medical students during their psychiatry clerkship aimed at teaching suicide risk assessment and safety planning. Our project is additive to the literature as a multimodal third-year medical student suicide risk assessment and safety planning workshop employing two SPs with suicidal ideation and measuring students’ self-perceived knowledge and comfort managing patients with suicidality. The target audience for our work is faculty developing medical student curricula that involve suicide risk assessment and safety planning.

## Methods

### Curriculum

**Figure 1.**
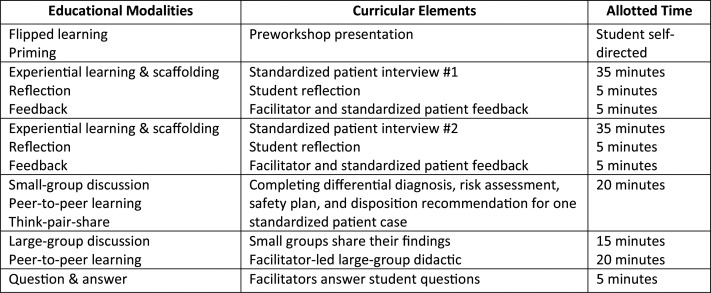
Workshop summary.

Third-year medical students participated in this required 2.5-hour in-person workshop (Figure 1) as part of their required 4-week psychiatry clerkship. The workshop included a preworkshop presentation, two SP encounters, small- and large-group exercises, and a large-group didactic. The workshop was conducted six times to accommodate all the psychiatry clerkship cohorts rotating in the first half of academic year 2023–2024. There were 15 to 21 students per cohort.

### Preworkshop Preparation

Each workshop required at least two facilitators and two SPs. The facilitators consisted of a psychiatry clerkship director and a volunteer attending psychiatrist or psychiatry resident physician. Facilitators did not receive formal training prior to the workshop but were encouraged to review the workshop educational materials in Appendices A–I. The authors provided guidance on facilitating SP encounters by offering a debriefing checklist, asking new facilitators to arrive early to review the workshop structure, and being available to answer questions and discuss expectations. Facilitators were knowledgeable in the subject area, as this is a foundational topic in psychiatry education.

The institution's SP program recruited the SPs for this workshop. They recruited SPs who matched the demographic factors of the case and possessed the acting skills necessary to accurately portray the scenarios. Four individuals, two for each case, were recruited to play the roles of Joe Jones (Appendix A) and Susan Olson (Appendix B). Scenarios were adapted from an unpublished educational activity previously used to train emergency medicine residents at our institution. Funding for the SPs came from our institution's psychiatry department.

Prior to the start of the academic year, SPs were trained during one of two (one per case) 1.5-hour sessions facilitated by a SP program leader and a project team member. During the training, the SP program leader reviewed the scenarios. A project team member answered questions during the training and answered additional SP questions before and after the simulations via email.

### Workshop Logistics

Prior to the workshop, learners were expected to view a presentation (Appendix C)^1,11–16^ covering session topics. Learners were not expected to have any prerequisite knowledge in the subject area prior to viewing this information. Expecting that learners would come into the workshop with different degrees of knowledge in the topic area, the authors created this preworkshop material as a primer.

SPs and facilitators arrived approximately 30 minutes prior to the session. Two separate spaces (room A and room B) were required to conduct the two SP scenarios simultaneously. This workshop took place at our institution's dedicated psychiatry residency space where rooms could be reserved without cost. Room A was large enough to comfortably fit the whole student cohort and both facilitators (just under 25 individuals maximum). This room had AV capability and a white board and was where the small- and large-group sessions occurred. Once students arrived, using the slides in Appendix D, facilitators divided learners into two equal groups. Half stayed in room A with a facilitator and Joe Jones, a man presenting to the emergency department with police for suicidal ideation in the setting of major depressive disorder, hazardous alcohol use, and grief. The other half of students went to room B (a smaller room that did not require AV capability or a white board and could hold just under 15 individuals maximum) to work with a facilitator and Susan Olson, a woman presenting to the emergency department voluntarily with contingent suicidal ideation in the setting of borderline personality disorder, alcohol use disorder, and chronic self-harm behaviors.

Prior to working with each of the SPs, students were given a door card with the patient's vital signs, brief presenting history, and minimal information obtained in triage (Appendices E and F). Students were instructed to take turns and each ask the SP one to three questions. Students were encouraged to assess for psychiatric concerns, review relevant history, determine risk and protective factors, and discuss aspects of a safety plan with the patient. The first 35 minutes were used for the SP interview and the final 10 minutes were used for student reflection and feedback from the facilitator and SP. Facilitator instructions and a debriefing checklist were included in the facilitator guide (Appendix G). Facilitators used the debriefing checklist to guide feedback while SPs provided natural, unscripted feedback. After the first 45-minute SP encounter, reflection, and feedback, students switched rooms so the SPs and facilitators could remain in the same room. Students were not graded during the SP interviews. A total of 1.5 hours was spent working with the SPs. After this time, the SPs were dismissed after being encouraged to discuss any other reactions with a project team member.

Students then reconvened in room A where, using the slides in Appendix D, facilitators reviewed suicide risk factors and protective factors. Students were then split into four small groups. Two groups were tasked with completing a differential diagnosis, risk assessment, safety plan, and disposition recommendation for Joe Jones, while two groups completed the same for Susan Olson. After this, students reconvened into a large group where small groups shared their findings. Facilitators wrote students’ thoughts on a white board, and, as part of a facilitator-led large-group didactic, facilitators compared students’ thoughts with those in Appendix D and provided additional information to guide students’ learning where necessary based on the group.

### Evaluation

To evaluate the effectiveness of the workshop in improving medical student self-assessment of knowledge and comfort managing patients with suicidality, all third-year medical student attendees were invited to participate in voluntary pre- and postworkshop surveys (Appendices H and I, respectively) created by the authors. The Medical College of Wisconsin Institutional Review Board approved this project prior to the 2023–2024 academic year. All students were known to be over age 18, eligible for the project, and notified about it at the beginning and end of the workshop (Appendix D). Surveys were disseminated via QR code included in the workshop slide deck (Appendix D). Project data were anonymously collected using Qualtrics, an online survey software that securely protects data and meets all applicable compliance regulations. There was no compensation for involvement, the decision to participate did not impact students’ grades, and survey completion was considered implied consent.

On the preworkshop survey (Appendix H), respondents were asked about prior formal suicide training and comfort managing patients with suicidality. They also indicated which aspects of suicide management they were most uncomfortable with. On both the pre- and postworkshop surveys (Appendices H and I), respondents indicated how knowledgeable they felt regarding assessing a patient with suicide and how comfortable they felt regarding managing a patient with suicide. It was considered that attaining educational objectives 1–5 would lead to improved student self-assessment of knowledge managing patients with suicidality. Lastly, on the postworkshop survey (Appendix I), respondents noted how relevant they found the course content, how helpful the SP encounters were, and what topics or questions they would have liked to see addressed.

### Data analysis

Data obtained during each workshop were collated and analyzed. Descriptive statistics as well as unpaired *t* tests (as surveys were unmatched) with *p* values (considered statistically significant if *p* < .05) and 95% confidence intervals, calculated using GraphPad, were utilized for data analysis and presentation.

## Results

**Figure 2.**
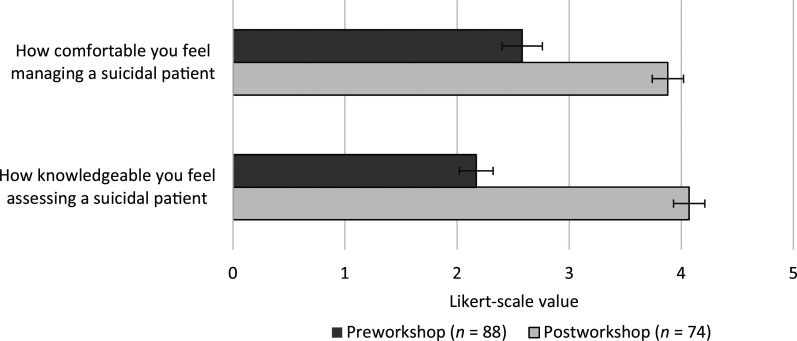
Improved knowledge and comfort managing patients with suicidality reported postworkshop. The question “How knowledgeable you feel assessing a suicidal patient?” was rated based on a 5-point Likert scale (1 = *not knowledgeable at all*, 5 = *extremely knowledgeable*). The question “How comfortable you feel managing a suicidal patient” was rated based on a 5-point Likert scale (1 = *not comfortable at all*, 5 = *extremely comfortable*). Error bars represent 95% confidence intervals of the means. For comparisons, *p* values were less than .001.

A total of 105 third-year medical students rotated through their psychiatry clerkship in the first half of the 2023–2024 academic year (July 2023 to December 2023) and attended the workshop. Of these 105 students, 88 (84%) completed the presurvey and 73 (70%) completed the postsurvey. All complete responses were analyzed. Prior to the workshop, most respondents 78% (69/88) reported no formal training managing patients with suicidality. Most respondents described their previous clinical experience managing patients with suicidality as *little experience*. On a 5-point Likert scale (1 = *not knowledgeable at all*, 5 = *extremely knowledgeable*), presurvey respondents rated their knowledge regarding the assessment of suicidality as 2.2. On a 5-point Likert scale (1 = *not comfortable at all*, 5 = *extremely comfortable*), presurvey respondents rated their comfort regarding the management of patients with suicidality as 2.6 (Figure 2). Respondents noted overseeing legal aspects, assessing level of risk, and determining final disposition as aspects of suicide management they felt most uncomfortable with. After the workshop, postsurvey respondents rated their knowledge regarding assessing patients with suicide as 4.1 and their comfort regarding the management of patients with suicide as 3.9. A statistically significant improvement in knowledge and comfort was observed pre- versus postworkshop (*p* < .001; Figure 2).

Using the same Likert scale (1 = *not at all*, 5 = *extremely*), respondents rated the workshop highly relevant (4.6) and helpful in illustrating best-practice concepts regarding the assessment and management of patients with suicidality (4.1). In the final open-ended question, respondents noted that they would appreciate further instruction regarding developing safety plans and deciding on final disposition in future sessions.

## Discussion

This suicide risk assessment and safety planning workshop improved medical students’ perceived knowledge and comfort managing suicidality. Educational techniques employed during this workshop included didactics, flipped learning, SP encounters, and group discussions. The required psychiatry clerkship provides an opportunity to address this important topic with all medical students. With calls for more required formal suicide training for medical students, this workshop appears to be an effective way of helping students feel more comfortable and knowledgeable managing suicidality.^17^

The authors intentionally developed a workshop that was multimodal, as research supports that medical students prefer multiple learning styles.^10^ Given that medical students matriculate with different clinical exposure and life experiences, the authors created a preworkshop presentation as a flipped-learning exercise and primer for the workshop. Though challenges have been identified with the use of primer exercises, including buy-in, particularly from medical students not going into the associated field of study,^18^ the authors still felt it was a potentially valuable exercise. In their study, Rose and colleagues used an online video primer activity before an in-class session and found an improvement in immediate and delayed posttest results, though the improvement was not statistically significant.^18^ The authors are not sure how many students in the present project completed the preworkshop primer, though efforts could be made to track this in the future.

The focal point of the workshop was the SP encounters. Suicidality can be difficult for students to assess due to a lack of knowledge about suicide, discomfort with the topic of suicide due to social taboos, and fear of negative outcomes. Though they can come with challenges in front-end preparation,^19^ SP encounters have been identified as ways for medical students to practice interviewing patients about sensitive topics and has been demonstrated as superior to peer interview, virtual reality, and learning with real patients in the clinical setting.^20^ SP simulations have also been demonstrated as effective approaches to teaching medical students about psychiatric risk assessments.^21^ Suicidality can also be difficult for SPs to discuss and portray. Our focus was on medical student and SP psychological safety. Future workshop trainings could include an emphasis on SP experiences and reactions.

During the SP encounters, for the interview technique, the authors chose to have students go around and each ask the SPs approximately one to three questions. This was to increase engagement by all students and to provide scaffolding for students who may not have been able to effectively complete the interview with the patient alone. SP encounters were designed to be a low-stakes, formative assessment so students would not have the pressure of receiving a grade but could receive feedback from peers, facilitators, and SPs.

Students felt the workshop was highly relevant, which may speak to medical students having some buy-in to the workshop topic. Furthermore, preworkshop findings from our project add to the limited data regarding medical students’ knowledge and comfort with suicidality preinterventions and can serve as a needs assessment for future curricula and educational activities in the subject area.

The authors studied the effectiveness of the workshop through pre- and postworkshop surveys. The workshop led to a statistically significant improvement in self-assessment of knowledge and comfort managing patients with suicidality (Figure 2). This improvement is consistent with data from a related study. Second-year medical student participants in the study by Chuop and colleagues reported improved familiarity and confidence with suicide prevention strategies, particularly suicide risk assessments and safety planning following a didactic and SP session.^6^ Other studies have found SP medical student experiences were well received but did not involve a patient with suicidal ideation at the time of assessment, unlike the two SPs in our workshop.^7,8^ A TBL experience by Lerchenfeldt and colleagues led to self-perceived solidifying of psychiatry skills and discussion about assessing for suicide risk factors.^9^

Our project is additive to the literature, as it involves a multimodal suicide risk assessment and safety planning workshop for third-year medical students and quantitatively measured students’ self-perceived knowledge and comfort managing a patient with suicidality (as both SPs in the workshop presented with suicidal ideation). Though statistically significant improvements were observed in our project, it is unknown if the perceived improvements are long lasting. To address this, authors could consider sending out a follow-up survey a period of time after the workshop.

Our project contains additional limitations not mentioned earlier in this section. Regarding the interview style of the SP encounters, the authors recognize that this may have led to a more scattered and less realistic interview than a one-on-one encounter. Additionally, the authors assessed students’ self-perceived improvement in knowledge and comfort managing suicidality. Future directions could include developing skills-based assessments, such as written risk assessments, written safety plans, or an OSCE at the end of the psychiatry clerkship, to evaluate for objective improvement managing suicidality postintervention. The long-term goal of this workshop is to teach students skills they can refine during the remainder of the psychiatry clerkship and, ultimately, continue to utilize as physicians in their eventual specialties.

## Appendices


SP Case - Joe Jones.docxSP Case - Susan Olson.docxPreworkshop Slides.pptxDidactic and Group Discussion Slides.pptxCase of Joe Jones Door Card.docxCase of Susan Olson Door Card.docxSP encounter Facilitator Guide.docxPreworkshop Survey.docxPostworkshop Survey.docx
*All appendices are peer reviewed as integral parts of the Original Publication.*
