# Supplementary material for: A Multimodal Workshop to Improve Medical Student Self-Assessment of Knowledge and Comfort Managing Patients With Suicidality
Source: MedEdPORTAL. 2025 Jan 17;21:11488. doi: 10.15766/mep_2374-8265.11488 (PMC11739282; doi:10.15766/mep_2374-8265.11488)
Supplement: Supplementary file 1 — SP Case - Joe Jones.docxSP Case - Susan Olson.docxPreworkshop Slides.pptxDidactic and Group Discussion Slides.pptxCase of Joe Jones Door Card.docxCase of Susan Olson Door Card.docxSP encounter Facilitator Guide.docxPreworkshop Survey.docxPostworkshop Survey.docx [file mep_2374-8265.11488-s001.zip › G. SP Encounter Facilitator Guide.docx]

**Appendix G. Facilitator Guide**

*There are 45 minutes allotted for each standardized patient encounter. The first 35 minutes are used for interviewing the standardized patient. Students should go around and ask the standardized patient 1-3 questions each. Encourage them to use this time to assess for the below items, including: psychiatric concerns, review relevant history, determine risk and protective factors, and discuss aspects of a safety plan with the patient. The last 10 minutes are for small group debriefing. In the first 5 minutes, students should reflect individually and then share in the group what aspects of the patient encounter they felt went well and which they feel could have been improved and how. In the second 5 minutes, facilitators and standardized patients are then asked to provide the same feedback to the students.*

| **Item** | **Example** |
| --- | --- |
| Greet patient, state name, and state role being clear and concise | “Hello, my name is student doctor _____. I am a third-year medical student who will be assisting in your care today. |
| Respect the patient’s personal space and be approachable | Keep an appropriate distance between you and the patient. Be calm and model approachable body language (i.e. face the patient). |
| Identify the patient’s chief concern and patient needs | “What brings you in today?” “How can we help you?” |
| Offer validation and reinforce that the patient is safe | “I understand there is a lot going on and you aren’t feeling your best.” “You’re at the hospital and are safe here.” |
| Explore precipitating factors contributing to the patient’s presentation | “What happened that brought you here?” “What do you think has led to this point?” |
| Identify static suicide risk factors | Obtain information about the patient’s gender, age, personal and family history of suicide attempts, personal history of psychiatric hospitalizations, recent loss, trouble with the law, and trauma history. To obtain the patient’s suicide history, ask questions such as: “Have you ever tried to end your life in the past?” “If so, how many attempts have you had?” “What was/were the method(s)?” “Were you hospitalized (medically and or psychiatrically) after?” |
| Identify dynamic suicide risk factors | Obtain information about any active substance use, psychiatric symptoms, engagement with outpatient mental health treatment, access to lethal means, employment status, and housing status. To obtain information about lethal means access, ask questions such as: “Do you have access to firearms?” “If so, how is/are the firearm(s) stored (i.e. locked, loaded, separate from ammunition)?” “Do you have access to large quantities of medications, or have you been collecting medications?” |
| Identify protective factors | Obtain information about social support(s), responsibility for family members and or pets, religious beliefs, future orientation, and help seeking behaviors. Appropriate questions could include: “What are some of your future goals?” and “what would you plan to do if you left the hospital today?” |
| Assess for current suicidal ideation and baseline suicidal thoughts (if applicable) | “Are you having any thoughts to end your life?” “Do you have a plan?” “Some individuals have a degree of suicidal thoughts at baseline, is this consistent with how you feel?” “How long have you had thoughts to end your life?” “What are you living for?” |
