# Supplementary material for: A Multimodal Workshop to Improve Medical Student Self-Assessment of Knowledge and Comfort Managing Patients With Suicidality
Source: MedEdPORTAL. 2025 Jan 17;21:11488. doi: 10.15766/mep_2374-8265.11488 (PMC11739282; doi:10.15766/mep_2374-8265.11488)
Supplement: Supplementary file 1 — SP Case - Joe Jones.docxSP Case - Susan Olson.docxPreworkshop Slides.pptxDidactic and Group Discussion Slides.pptxCase of Joe Jones Door Card.docxCase of Susan Olson Door Card.docxSP encounter Facilitator Guide.docxPreworkshop Survey.docxPostworkshop Survey.docx [file mep_2374-8265.11488-s001.zip › H. Preworkshop Survey.docx]

**Appendix H. Suicide Risk Assessment and Safety Planning Workshop Preworkshop Survey**

*If desired, this optional preworkshop survey can be distributed to medical students.*

1. What month of your clerkship are you currently in?

- July – September
- October – December
- January – March
- April – June

1. Have you had any prior formal training in managing suicidal patients (prior to the pre-workshop material)?

- Yes
- No

1. If yes, what type of training have you received? Choose all that apply.

- Didactic presentation(s) on suicide risk assessment
- OSCE-style case simulations
- Topical conference presentation attendance
- Other

1. If other, please describe.
2. How much clinical experience have you previously had in managing suicidal patients?

- Significant experience
- Moderate experience
- Some experience
- Little experience
- No prior experience

1. How knowledgeable do you feel regarding the assessment of suicidal patients?

- Not knowledgeable at all
- Slightly knowledgeable
- Moderately knowledgeable
- Very knowledgeable
- Extremely knowledgeable

1. How comfortable do you feel regarding the management of suicidal patients?

- Not comfortable at all
- Somewhat uncomfortable
- Neither comfortable nor uncomfortable
- Somewhat comfortable
- Extremely comfortable

1. With which aspects of the management of suicidal patients are you most uncomfortable? Choose all that apply.

- Assessing level of risk (e.g., imminent, acute, chronic, etc.)
- Maintaining patient safety in the clinical setting
- Determination of final disposition
- Overseeing legal aspects
- Interdisciplinary team management
- Documentation
- When to call a psychiatry consult
- Developing a safety plan
