# Supplementary material for: A Multimodal Workshop to Improve Medical Student Self-Assessment of Knowledge and Comfort Managing Patients With Suicidality
Source: MedEdPORTAL. 2025 Jan 17;21:11488. doi: 10.15766/mep_2374-8265.11488 (PMC11739282; doi:10.15766/mep_2374-8265.11488)
Supplement: Supplementary file 1 — SP Case - Joe Jones.docxSP Case - Susan Olson.docxPreworkshop Slides.pptxDidactic and Group Discussion Slides.pptxCase of Joe Jones Door Card.docxCase of Susan Olson Door Card.docxSP encounter Facilitator Guide.docxPreworkshop Survey.docxPostworkshop Survey.docx [file mep_2374-8265.11488-s001.zip › I. Postworkshop Survey.docx]

**Appendix I. Suicide Risk Assessment and Safety Planning Workshop Postworkshop Survey**

*If desired, this optional postworkshop survey can be distributed to medical students.*

1. What month of your clerkship are you currently in?

- July – September
- October – December
- January – March
- April – June

1. Rate the overall relevance of the subject matter or course content.

- Extremely relevant
- Very relevant
- Moderately relevant
- Slightly relevant
- Not relevant at all

1. After this learning experience, how knowledgeable do you feel regarding the evaluation and management of suicide risk?

- Extremely knowledgeable
- Very knowledgeable
- Moderately knowledgeable
- Slightly knowledgeable
- Not knowledgeable at all

1. After this learning experience, how knowledgeable do you feel in assessing a suicidal patient?

- Extremely knowledgeable
- Very knowledgeable
- Moderately knowledgeable
- Slightly knowledgeable
- Not knowledgeable at all

1. After this learning experience, how comfortable do you feel in managing a suicidal patient, including developing a safety plan?

- Extremely comfortable
- Somewhat comfortable
- Neither comfortable nor uncomfortable
- Somewhat comfortable
- Not comfortable at all

1. How helpful was the patient case simulation in illustrating the best-practice concepts related to evaluating and managing suicidal patients?

- Extremely helpful
- Very helpful
- Moderately helpful
- Slightly helpful
- Not helpful at all

1. What topics or questions would you have liked to see addressed (related to suicidal patients) that were not covered today? Please describe.
