# Supplementary material for: A Multimodal Workshop to Improve Medical Student Self-Assessment of Knowledge and Comfort Managing Patients With Suicidality
Source: MedEdPORTAL. 2025 Jan 17;21:11488. doi: 10.15766/mep_2374-8265.11488 (PMC11739282; doi:10.15766/mep_2374-8265.11488)
Supplement: Supplementary file 1 — SP Case - Joe Jones.docxSP Case - Susan Olson.docxPreworkshop Slides.pptxDidactic and Group Discussion Slides.pptxCase of Joe Jones Door Card.docxCase of Susan Olson Door Card.docxSP encounter Facilitator Guide.docxPreworkshop Survey.docxPostworkshop Survey.docx [file mep_2374-8265.11488-s001.zip › A. SP Case - Joe Jones.docx]

Appendix A. SP Case – Joe Jones

*Distributed to standardized patient (SP) and facilitator during training. This portion of the workshop is expected to take a total of 45 minutes. This includes 35 minutes for SP interview, 5 minutes for medical student reflection, and 5 minutes for facilitator and SP feedback.*

Date: May 19, 2023

Case Authors: Deb Kennedy, MD, Paige Chardavoyne, MD, MEd, Julie Owen, MD, MBA

Name of Case: Case of Joe Jones

Name of Educational and/or Assessment Activity: A multimodal workshop to improve medical student knowledge and comfort managing patients with suicidality

Patient Name: Joe Jones

Chief Complaint: “I want to die!”

Most Likely Diagnosis and Differential with Rationale From History and/or Physical Exam:

- Major depressive disorder - leading diagnosis. Patient with greater than 2 months of depressive symptoms including lower mood, sleep changes (trouble staying asleep), appetite changes (25# weight loss in the last 6 months), poorer concentration, anhedonia, feelings of guilt, and suicidal ideation.
- Alcohol use disorder – patient reports drinking ½ of a pint of vodka daily currently. Reported regular alcohol use for the last 2 months. Further information is needed to assess for alcohol use disorder and, if present, its severity. As it is not the focus of this case, if the SP is asked further details about his alcohol use, he can state “I don’t want to talk about that right now”.
- Substance-induced mood disorder – less likely as patient’s symptoms of major depressive disorder predates his regular alcohol use
- Prolonged grief disorder – less likely as patient meeting criteria for major depressive disorder

Challenge Question: What do you feel would be the most appropriate disposition for Joe Jones based on today’s encounter?

Domains: Check all that apply

- Professionalism

X Communication and Interpersonal Skills

X Medical History

- Physical Exam
- Shared Decision-Making
- Patient Education

X Clinical Reasoning

- Documentation
- Handoff
- Presentation
- Other:

Type and Level of Learner: Third-year medical student

Case Objectives: Please list specific objectives for each of the domains you have checked above:

Communication and interpersonal skills:

1. Develop a therapeutic alliance with a patient.

2. Use active listening skills and provide support to a patient presenting with acute psychiatric symptoms.

3. Effectively identify and discuss safety concerns with a patient.

Medical history:

1. Complete a psychiatric review of symptoms.

2. Obtain other relevant medical history.

3. Gain information necessary to complete a risk assessment for a patient.

Clinical reasoning:

1. Develop a differential diagnosis for a patient.

2. Create a suicide risk assessment for a patient.

3. Identify aspects of a safety plan for a patient.

4. Make a disposition recommendation for a patient based on diagnosis, risk assessment, and safety plan.

| SETTING: outpatient, in patient, ED, home, nursing home, rehab, group, etc. | Emergency department |
| --- | --- |
| PATIENT PROFILE: Information about the “patient” that helps select an SP and helps the learner get an understanding of them as a person. SP will know more information about the patient than learner will ever ask but allows SP to portray a fully developed patient personality. If none of the items below are particulars for the case, please write “all may be used.” | |
| Age range | 60s |
| Religious/spiritual background | All may be used |
| Sex (e.g., male, female, intersex, transwoman, transman) | Male |
| Sexual orientation (e.g., heterosexual, lesbian, gay, bisexual, pansexual, queer, asexual) | Heterosexual |
| Gender expression (e.g., man, woman, genderqueer) | Male |
| Race and ethnicity | All may be used |
| Physical description (e.g., BMI, height range) | All may be used |
| Physical limitations | All may be used |
| Patient appearance (e.g., disheveled, hospital gown, business casual, casual) | Slightly disheveled, casual |
| Moulage + location (e.g., none, bruises, scars, body piercing, tattoos) | None |
| Affect (e.g., pleasant, cooperative) | Depressed, guarded |
| Family group (e.g., who is family, who they live with) | Lives alone since his wife passed away. Has one close friend. Has some family supports but has not been in contact with them recently. |
| Education | Graduated high school |
| Level of health literacy | Average |
| Employment, if any - present and past, noting any current stresses | Retired from US military. Previously was in combat in Vietnam. |
| Home/homeless - type of dwelling, number of stories, owned or rented | Lives alone in an apartment he rents |
| Financial situation - any current stresses | Service connected for PTSD from the VA. No current financial stress. |
| Insurance status (e.g., un/under/insured, public/private, HMO/PPO) | Public (Tricare/VA benefits) |
| Habits (i.e., diet, exercise, caffeine, smoking, alcohol, drugs) | Smokes nicotine daily and has been recently drinking ½ of a pint of vodka daily. If asked about impact alcohol has had on his life or other screening questions for alcohol use disorder, patient can state “I don’t want to talk about that right now”. No other substance use reported |
| Activities (i.e., hobbies, sports, clubs, friends) | None |
| Typical day - what is the usual daily routine | Stays at home most of the day, does not get out much, isolated. |

| CASE INFORMATION | |
| --- | --- |
| Chief Concern: What the patient will say when greeted by the student. The patient’s primary reason for seeking medical care often stated in their own words. | “I want to die!” |
| Additional Concerns: Other, if any, concerns the patient has today (i.e., symptoms, requests, expectations, etc.) that will become part of set agenda. | The patient texted his friend today that he plans to shoot himself. Patient’s friend called police for a welfare check. Police brought the patient to the emergency department. |
| THE PATIENT’S STORY: The SP will be asked to tell their symptom story and the personal and emotion impact for each of their concerns. You will want to write this in the patient’s voice. The symptom story should be able to answer this question: “Tell me more about [chief concern/additional concern], starting at the beginning and bringing me up to now.”  The personal context should be able to answer questions concerning the broader personal/psychosocial context of symptoms, especially the patient’s beliefs/attributions.  The emotional context should be able to ask how are you doing with this, how does this make you feel, how has this affected you emotionally? IMPACT: How has this affected your life? How has this been for your family? | - Patient has been experiencing depressive symptoms since his wife passed away 6 months ago. Specifically, he has been experiencing lower mood, sleep changes (trouble staying asleep), appetite changes (25# weight loss in the last 6 months), poorer concentration, anhedonia, feelings of guilt, and suicidal ideation. - Associated symptoms include hearing his wife’s voice asking him to be with her and wishing he was with his wife. Not currently having PTSD-related nightmares - Two months ago he began drinking alcohol regularly and he is now drinking ½ of a pint of vodka daily. - He was prescribed escitalopram 10 mg daily by his primary care provider at the VA five months ago but has not been taking it and has not followed up. - Has access to a firearm at home - Has no allergies and past medical history is only significant for PTSD and hypertension. |
| HISTORY OF PRESENT ILLNESS: Although some of the HPI will be given in the patient’s symptom story, the learners will expand the story during the direct question section. Below, describe the detailed history, usually about the chief concern, which the student must develop in order to make a useful assessment of the problem: | |
| Onset (when; gradual or sudden) | Symptoms began after the death of his wife 6 months ago and have gradually worsened. Three months ago, he began having suicidal ideation without plan or intent. Within the last week, has developed a plan to shoot himself with a firearm he owns with the intent to end his life. |
| Setting (what was going on or where was patient when symptoms first noticed?) | Home |
| Duration (how long) | Depressive symptoms for the last 6 months |
| Time relationships (frequency, constant or intermittent) | Constant |
| Location | Psychiatric |
| Radiation | Not applicable |
| Quality | Not applicable |
| Amount | Not applicable |
| Aggravated by what | Special occasions (such as his late wife’s birthday), holidays, and hearing his late wife’s voice |
| Relieved by what | Talking with his friend |
| Associated with what | Auditory hallucination hearing his wife’s voice |
| Attitude (what does the patient think is the problem, and how do they feel about it) | Depressed, guarded, intermittently tearful |
| Overall course | Presence of depressive symptoms for the last 6 months with worsening and onset of suicidal ideation (now with plan and intent) |
| REVIEW OF SYSTEMS: Significant positives and negatives | |
| Psychiatric | - Active suicidal ideation with plan to shoot himself with a firearm he owns and intent to end his life - Having auditory hallucinations hearing his wife’s voice - Denies homicidal ideation and visual hallucinations - Recently decreased appetite (25# weight loss in last 6 months), poorer energy, poorer concentration, lower mood - Not currently having PTSD-related nightmares - No symptoms of mania reported - No concerns about body image |
| Medical | No additional symptoms reported. No symptoms of alcohol withdrawal reported. |
| Past medical history |  |
| Medication allergies (name and reaction) | None |
| Environmental allergies (name and reaction) | None |
| Illnesses | None |
| Vaccinations | Fully vaccinated |
| Surgeries | None |
| Accidents/injuries/trauma | None |
| Hospitalization | None |
|  | |
| Inclusive sexual and reproductive history | |
| Sexual practices  Sexual partners  Protection: Use of safer sex practices  Use of birth control if appropriate  Risk of intimate partner violence | Heterosexual, 3 lifetime partners.  Not sexually active since his wife passed away and had not been sexually active with anyone else besides her for 30 years |
| OB/GYN history | Not applicable |
| Medications | Prescription/dose/reason: amlodipine 10 mg daily for hypertension (intermittently adherent) and escitalopram 10 mg daily for depression (prescribed 5 months ago, stopped taking it several months ago because “I didn’t want to take more medication and I’m not sure it was helping”)  Over the counter/dose/reason: none  Herbs/supplements/dose/reason: none  Other: none |
| Immunizations | Fully immunized |
| Tobacco products:  X Cigarettes   - Cigar - Pipe - Chew - E-cigarettes | - Never - Past - year started/year quit   X Current   - - Quantity: ½ pack per day   - # of years: 25 years |
| Alcohol   - Beer - Wine   X Liquor   - Other | - Never - Past - year started/year quit   X Current   - - Quantity: ½ pint of vodka daily   - # of years: He has been drinking alcohol daily for 2 months |
| Drugs   - Marijuana - Cocaine - Heroin - Methamphetamine - IV drug use - Inhalants - Other | X Never   - Past - year started/year quit - Current   - Quantity   - # of years |
| Diet (describe) | All may be used |
| Exercise (describe) | All may be used |
| List any other important social history or information important to this case | Not applicable |
| Family history |  |
| Mother, father, siblings, grandparents, and other significant findings | Major depressive disorder (mother), alcohol use disorder (father), and non-fatal suicide attempt (cousin) |
|  |  |
| Physical Exam - List exam maneuvers expected for this case and any abnormal findings that SP will simulate. (tenderness, hyper-hypo reflex, rebound, weakness, etc.)  Not applicable. The patient will not allow the interviewer to perform a physical exam due to irritability. The patient should state “don’t come near me” if interviewer attempts to approach patient for a physical exam. | |
| PHYSICAL EXAM FINDINGS |  |
| 1. Written in layperson’s terms |  |
| 1. General appearance - affect, appearance, position of patient at opening (i.e., sitting, lying down, holding abdomen, etc.) | Slightly disheveled, seated, slower movements, slower speech with low volume, minimal eye contact, “depressed” mood with mood-congruent and blunted affect. |
| 1. Vital signs | BP 155/90, HR 85, SpO2 100% on room air, RR 15 |
| 1. Specific findings and affect | Becomes tearful when talking about his wife. Expresses plan to shoot himself in an attempt to end his life after leaving the hospital. No signs of alcohol withdrawal on assessment. |
| 1. Response to certain physical movements | Not applicable |
|  |  |
| DIAGNOSIS AND DIFFERENTIAL |  |
| Diagnosis with support from positive and negative history and PE findings | Major depressive disorder - leading diagnosis. Patient with greater than 2 months of depressive symptoms including lower mood, sleep changes (trouble staying asleep), appetite changes (25# weight loss in the last 6 months), poorer concentration, anhedonia, feelings of guilt, and suicidal ideation. |
| Differential with support from positive and negative history and PE findings | - Alcohol use disorder – patient reports drinking ½ of a pint of vodka daily currently. Reported regular alcohol use for the last 2 months. Further information is needed to assess for alcohol use disorder and, if present, its severity. As it is not the focus of this case, if the SP is asked further details about his alcohol use, he can state “I don’t want to talk about that right now”. - Substance-induced mood disorder – less likely as patient’s symptoms of major depressive disorder predates his regular alcohol use - Prolonged grief disorder – less likely as patient meeting criteria for major depressive disorder |
|  |  |
| MANAGEMENT OR DIAGNOSTIC PLAN | - Disposition: inpatient psychiatric hospitalization for psychiatric stabilization (including medication management) - Though the safety plan would likely be made on the inpatient unit, consider some aspects of the patient’s safety plan, including limiting access to lethal means (removing the firearm from the home until depression and suicidal ideation resolve) and utilizing support (primary care clinic, family, friend), including crisis resources, when needed. |
|  |  |
| PROFESSIONALISM ISSUES OR CHALLENGES | - Challenges establishing rapport with the patient who presents very depressed and guarded. - Appropriate support should be provided to the patient throughout the interview. |
