# Supplementary material for: A Multimodal Workshop to Improve Medical Student Self-Assessment of Knowledge and Comfort Managing Patients With Suicidality
Source: MedEdPORTAL. 2025 Jan 17;21:11488. doi: 10.15766/mep_2374-8265.11488 (PMC11739282; doi:10.15766/mep_2374-8265.11488)
Supplement: Supplementary file 1 — SP Case - Joe Jones.docxSP Case - Susan Olson.docxPreworkshop Slides.pptxDidactic and Group Discussion Slides.pptxCase of Joe Jones Door Card.docxCase of Susan Olson Door Card.docxSP encounter Facilitator Guide.docxPreworkshop Survey.docxPostworkshop Survey.docx [file mep_2374-8265.11488-s001.zip › B. SP Case - Susan Olson.docx]

Appendix B. SP Case – Susan Olson

*Distributed to standardized patient (SP) and facilitator during training. This portion of the workshop is expected to take a total of 45 minutes. This includes 35 minutes for SP interview, 5 minutes for medical student reflection, and 5 minutes for facilitator and SP feedback.*

Date: May 19, 2023

Case Authors: Deb Kennedy, MD, Paige Chardavoyne, MD, MEd, Julie Owen, MD, MBA

Name of Case: Case of Susan Olson

Name of Educational and/or Assessment Activity: A multimodal workshop to improve medical student knowledge and comfort managing patients with suicidality

Patient Name: Susan Olson

Chief Complaint: “I want to die!”

Most Likely Diagnosis and Differential with Rationale From History and/or Physical Exam:

- Borderline personality disorder - leading diagnosis. Patient with history of chronic passive suicidal ideation, self-harm, impulsive behavior, low self-esteem, and concerns with abandonment
- Substance (alcohol)-induced mood disorder: patient with depressed mood and suicidal thoughts in the setting of consistent alcohol use (though more today). However, few clear depressive symptoms noted/reported.
- Unspecified mood disorder - rule out major depressive disorder, adjustment disorder with depressed mood, and bipolar disorder: patient with mood symptoms requiring further diagnostic clarification (ie, number of depressive symptoms/their time course and further characterization of mood lability to rule out hypomanic/manic episodes)

Challenge Question: What do you feel would be the most appropriate disposition for Susan Olson based on today’s encounter?

Domains: Check all that apply

- Professionalism

X Communication and Interpersonal Skills

X Medical History

- Physical Exam
- Shared Decision-Making
- Patient Education

X Clinical Reasoning

- Documentation
- Handoff
- Presentation
- Other:

Type and Level of Learner: Third-year medical student

Case Objectives: Please list specific objectives for each of the domains you have checked above:

Communication and interpersonal skills:

1. Develop a therapeutic alliance with a patient.

2. Use active listening skills and provide support to a patient presenting with acute psychiatric symptoms.

3. Effectively identify and discuss safety concerns with a patient.

Medical history:

1. Complete a psychiatric review of symptoms.

2. Obtain other relevant medical history.

3. Gain information necessary to complete a risk assessment for a patient.

Clinical reasoning:

1. Develop a differential diagnosis for a patient.

2. Create a suicide risk assessment for a patient.

3. Identify aspects of a safety plan for a patient.

4. Make a disposition recommendation for a patient based on diagnosis, risk assessment, and safety plan.

| SETTING: outpatient, in patient, ED, home, nursing home, rehab, group, etc. | Emergency department |
| --- | --- |
| PATIENT PROFILE: Information about the “patient” that helps select an SP and helps the learner get an understanding of them as a person. SP will know more information about the patient than learner will ever ask but allows SP to portray a fully developed patient personality. If none of the items below are particulars for the case, please write “all may be used.” | |
| Age range | 20-35 |
| Religious/spiritual background | All many be used |
| Sex (e.g., male, female, intersex, transwoman, transman) | Female |
| Sexual orientation (e.g., heterosexual, lesbian, gay, bisexual, pansexual, queer, asexual) | All may be used |
| Gender expression (e.g., man, woman, genderqueer) | All may be used |
| Race and ethnicity | All may be used |
| Physical description (e.g., BMI, height range) | All may be used |
| Physical limitations | All may be used |
| Patient appearance (e.g., disheveled, hospital gown, business casual, casual) | Casual, fair grooming |
| Moulage + location (e.g., none, bruises, scars, body piercing, tattoos) | Laceration on forearm |
| Affect (e.g., pleasant, cooperative) | Tearful, irritable |
| Family group (e.g., who is family, who they live with) | Lives with parents and younger sister |
| Education | Started but did not complete first year of college at a local university about 1 year ago |
| Level of health literacy | Average |
| Employment, if any - present and past, noting any current stresses | Unemployed and does not want to find a job at this time |
| Home/homeless - type of dwelling, number of stories, owned or rented | Family owns the home where the patient lives with parents and younger sister |
| Financial situation - any current stresses | Currently unemployed and receives financial support from parents |
| Insurance status (e.g., un/under/insured, public/private, HMO/PPO) | Medicaid |
| Habits (i.e., diet, exercise, caffeine, smoking, alcohol, drugs) | Smokes cigarettes daily, uses marijuana on the weekends, and drinks alcohol daily |
| Activities (i.e., hobbies, sports, clubs, friends) | Enjoys playing video games and spending time with boyfriend |
| Typical day - what is the usual daily routine | Wakes up around noon, spends significant time isolated in bedroom, plays video games during the day, frequently talking (ang arguing) with boyfriend on phone, stays up late at night |

| CASE INFORMATION | |
| --- | --- |
| Chief Concern: What the patient will say when greeted by the student. The patient’s primary reason for seeking medical care often stated in their own words. | “I want to die!” |
| Additional Concerns: Other, if any, concerns the patient has today (i.e., symptoms, requests, expectations, etc.) that will become part of set agenda. | Patient presents voluntarily to the ED |
| THE PATIENT’S STORY: The SP will be asked to tell their symptom story and the personal and emotion impact for each of their concerns. You will want to write this in the patient’s voice. The symptom story should be able to answer this question: “Tell me more about [chief concern/additional concern], starting at the beginning and bringing me up to now.”  The personal context should be able to answer questions concerning the broader personal/psychosocial context of symptoms, especially the patient’s beliefs/attributions.  The emotional context should be able to ask how are you doing with this, how does this make you feel, how has this affected you emotionally? IMPACT: How has this affected your life? How has this been for your family? | - Patient presents with acute suicidal ideation following a fight with her boyfriend. Has chronic passive suicidal ideation. They have been dating for 2 months. The patient is concerned he will end the relationship and feels “he is the right one”. This is the patient’s fifth boyfriend in the last two years. Of these, the longest relationship was 6 months. Feels as though her acute suicidal ideation would resolve if she were able to reach her boyfriend via phone to provide reassurance that he will not leave her. - During periods of increased stress, has a history of intermittent auditory hallucinations in the form of “hearing whispers” and visual hallucinations in the form of “seeing shadows”. - History of suicide attempt at age 19 via overdose on ibuprofen when her mother did not let her travel abroad with friends. Told her mother about the ingestion immediately after. Was taken to a medical hospital and then transferred to a psychiatric hospital. - History of self-harm in the form of cutting forearms when angry or upset. Several scars are present on bilateral forearms. - Patient has a history of physical and verbal abuse from boyfriends and a history of sexual trauma at age 13. - Was prescribed escitalopram 10 mg daily for depression by a psychiatrist 3 months ago. Stopped taking it and stopped following up a couple months ago because “it does not help”. Has trialed several antidepressant medications in the past but does not feel these were helpful. - No access to firearms but does have scissors and knives at home. - Drank a pint of vodka before presenting to the emergency department tonight. - No other medical history (aside from history of depression, bulimia nervosa, and alcohol use disorder) and no allergies. |
| HISTORY OF PRESENT ILLNESS: Although some of the HPI will be given in the patient’s symptom story, the learners will expand the story during the direct question section. Below, describe the detailed history, usually about the chief concern, which the student must develop in order to make a useful assessment of the problem: | |
| Onset (when; gradual or sudden) | Sudden onset active suicidal ideation after a fight with her boyfriend. |
| Setting (what was going on or where was patient when symptoms first noticed?) | Home |
| Duration (how long) | Active suicidal ideation began within the last couple hours. |
| Time relationships (frequency, constant or intermittent) | Passive suicidal ideation and self-harm behaviors/urges are chronic. |
| Location | Not applicable |
| Radiation | Not applicable |
| Quality | Not applicable |
| Amount | Not applicable |
| Aggravated by what | Interpersonal conflict, concerns she will be abandoned, alcohol use/intoxication |
| Relieved by what | Self-harm, reassurance |
| Associated with what | Seeing shadows and hearing whispers |
| Attitude (what does the patient think is the problem, and how do they feel about it) | Patient is tearful, anxious, irritable. Believes the problem is that her boyfriend will leave her. Believes her symptoms would resolve if she knew he was not going to leave her |
| Overall course | Acute on chronic psychiatric symptoms |
| REVIEW OF SYSTEMS: Significant positives and negatives | |
| Psychiatric | - Chronic low mood, chronic intermittent insomnia, intermittent feelings of hopelessness (particularly following interpersonal conflict), feelings of emptiness. - Chronic suicidal ideation and self-harm urges/behaviors (cutting). - History of bulimia nervosa at age 15. No current changes to appetite/weight and no current disordered eating. - Auditory hallucinations in the form of “hearing whispers” and visual hallucinations in the form of “seeing shadows” now and in periods of increased stress. No other psychotic symptoms. - No history of or current symptoms of mania. |
| Medical | No additional symptoms reported. No signs or symptoms of alcohol withdrawal reported. |
| Past medical history |  |
| Medication allergies (name and reaction) | None |
| Environmental allergies (name and reaction) | None |
| Illnesses | None |
| Vaccinations | Fully vaccinated |
| Surgeries | None |
| Accidents/injuries/trauma | Chronic self-harm in the form of cutting. Has several scars from lacerations to her bilateral forearms. One new laceration present from prior to arrival. |
| Hospitalization | History of psychiatric hospitalization following suicide attempt at age 19. History of several other psychiatric hospitalizations for worsening depression and suicidal ideation. |
|  | |
| Inclusive sexual and reproductive history | |
| Sexual practices  Sexual partners  Protection: Use of safer sex practices  Use of birth control if appropriate  Risk of intimate partner violence | History of several sexual partners |
| OB/GYN history | Not applicable |
| Medications | Prescription/dose/reason: Most recently prescribed escitalopram 10 mg daily for depression but stopped taking a couple months ago. Has been on other antidepressant medications in the past but does not recall their names  Over the counter/dose/reason: None  Herbs/supplements/dose/reason: None  Other: None |
| Immunizations | Fully vaccinated |
| Tobacco products:  X Cigarettes   - Cigar - Pipe - Chew - E-cigarettes | - Never - Past - year started/year quit   X Current   - - Quantity: ½ pack per day   - # of years: 5 years |
| Alcohol   - Beer - Wine   X Liquor   - Other | - Never - Past - year started/year quit   X Current   - - Quantity: ½ pint per day of vodka   - # of years: 6 months |
| Drugs  X Marijuana   - Cocaine - Heroin - Methamphetamine - IV drug use - Inhalants - Other | - Never - Past - year started/year quit   X Current   - - Quantity: 1 bowl on weekends   - # of years: 3 years |
| Diet (describe) | All may be used |
| Exercise (describe) | Minimal exercise, prefers to be inside playing video games |
| List any other important social history or information important to this case | Not applicable |
| Family history |  |
| Mother, father, siblings, grandparents, and other significant findings | Major depressive disorder (mother), alcohol use disorder (father), and non-fatal suicide attempt (cousin) |
|  |  |
| Physical Exam - List exam maneuvers expected for this case and any abnormal findings that SP will simulate. (tenderness, hyper-hypo reflex, rebound, weakness, etc.)  Not applicable. Patient will not allow interviewer to perform a physical exam due to irritability. Patient should state “don’t come near me” if interviewer attempts to approach patient for a physical exam. | |
| PHYSICAL EXAM FINDINGS |  |
| 1. Written in layperson’s terms |  |
| 1. General appearance - affect, appearance, position of patient at opening (i.e., sitting, lying down, holding abdomen, etc.) | Dressed casually, seated, no abnormal movements, intermittent eye contact. Normal volume initially then begins to raise voice as interview progresses. Has laceration on one forearm that will not require stitches. Expresses suicidal ideation with plan to cut her wrists in an attempt to end her life if she is unable to reach her boyfriend via phone and hear him tell her he will not leave her. |
| 1. Vital signs | BP 120/75, HR 80, SpO2 100% on room air, RR 18 |
| 1. Specific findings and affect | Tearful, irritable, begins to raise voice as interview progresses. No signs or symptoms of alcohol withdrawal on assessment. |
| 1. Response to certain physical movements | Patient should not approach or threaten interviewer or threaten to leave against medical advice. |
|  |  |
| DIAGNOSIS AND DIFFERENTIAL |  |
| Diagnosis with support from positive and negative history and PE findings | Borderline personality disorder - leading diagnosis. Patient with history of chronic passive suicidal ideation, self-harm, impulsive behavior, low self-esteem, and concerns with abandonment |
| Differential with support from positive and negative history and PE findings | - Substance (alcohol)-induced mood disorder: patient with depressed mood and suicidal thoughts in the setting of consistent alcohol use (though more today). However, few clear depressive symptoms noted/reported. - Unspecified mood disorder - rule out major depressive disorder, adjustment disorder with depressed mood, and bipolar disorder: patient with mood symptoms requiring further diagnostic clarification (ie, number of depressive symptoms/their time course and further characterization of mood lability to rule out hypomanic/manic episodes) |
|  |  |
| MANAGEMENT OR DIAGNOSTIC PLAN | - Complete a safety plan with the patient, including: limiting access to lethal means (which could include locking up medications and sharps), increasing family supervision, and utilizing supports, including crisis resources, when needed. - Disposition: home considering higher level of care such as partial hospitalization program (PHP) or intensive outpatient program (IOP) |
|  |  |
| PROFESSIONALISM ISSUES OR CHALLENGES | - Challenges establishing rapport with the patient. - Appropriate support should be provided to the patient throughout the interview. |
