# Supplementary material for: A Multimodal Workshop to Improve Medical Student Self-Assessment of Knowledge and Comfort Managing Patients With Suicidality
Source: MedEdPORTAL. 2025 Jan 17;21:11488. doi: 10.15766/mep_2374-8265.11488 (PMC11739282; doi:10.15766/mep_2374-8265.11488)
Supplement: Supplementary file 1 — SP Case - Joe Jones.docxSP Case - Susan Olson.docxPreworkshop Slides.pptxDidactic and Group Discussion Slides.pptxCase of Joe Jones Door Card.docxCase of Susan Olson Door Card.docxSP encounter Facilitator Guide.docxPreworkshop Survey.docxPostworkshop Survey.docx [file mep_2374-8265.11488-s001.zip › C. Preworkshop Slides.pptx]

## Slide 1
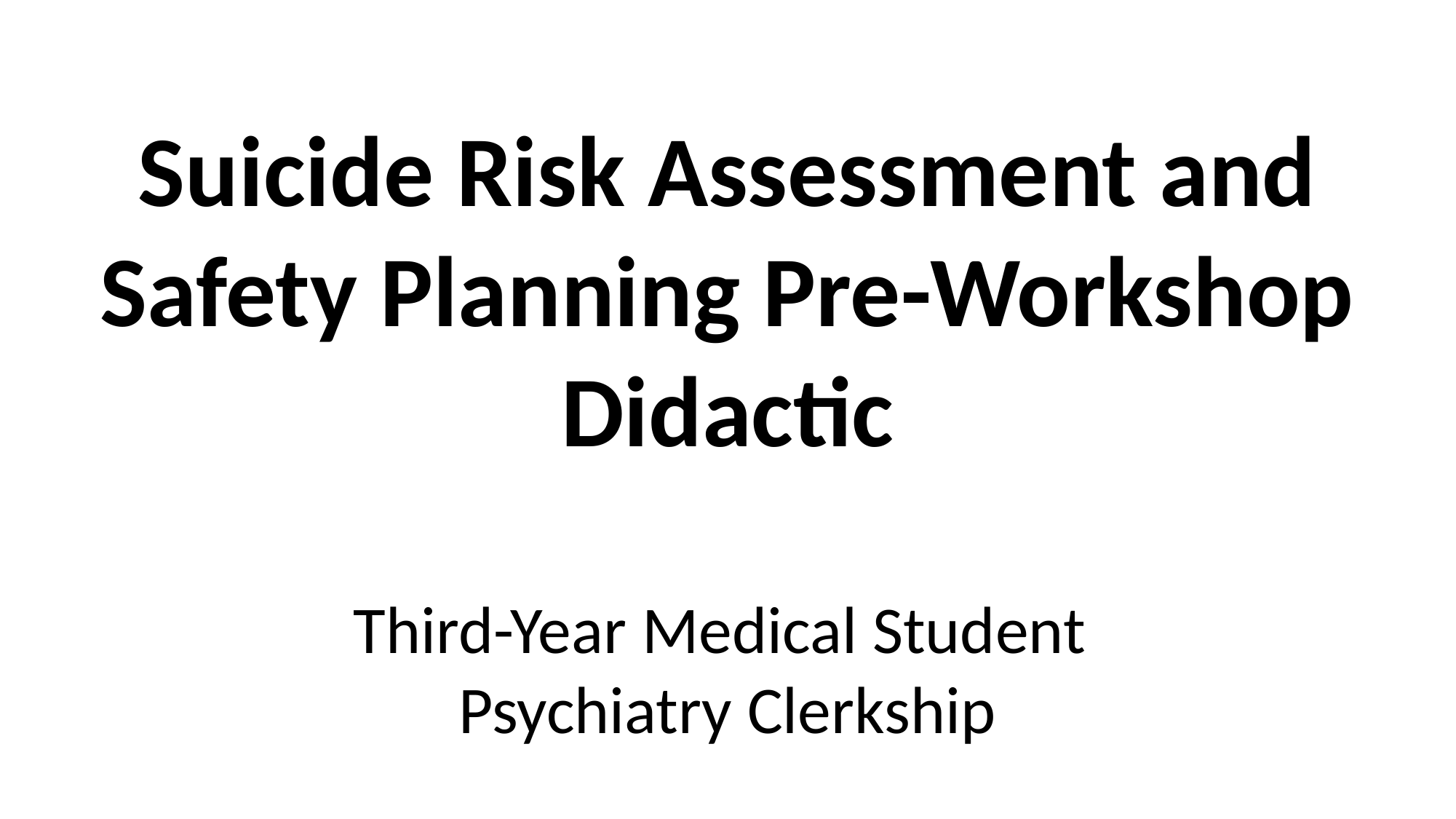

Suicide Risk Assessment and Safety Planning Pre-Workshop Didactic
Third-Year Medical Student
Psychiatry Clerkship
# Assessment and Management of the Suicidal Patient

## Slide 2
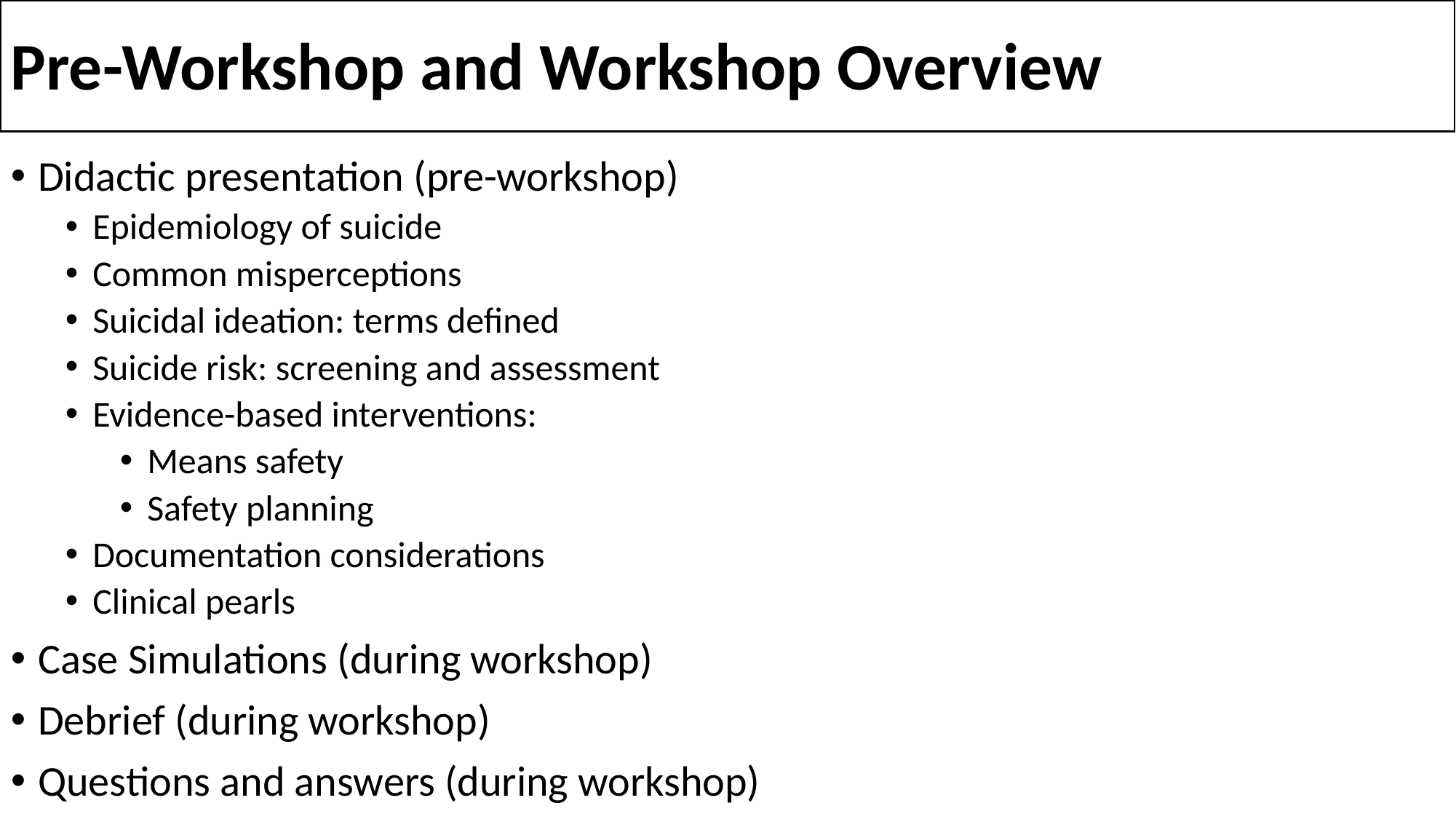

Pre-Workshop and Workshop Overview
Didactic presentation (pre-workshop)
Epidemiology of suicide
Common misperceptions
Suicidal ideation: terms defined
Suicide risk: screening and assessment
Evidence-based interventions:
Means safety
Safety planning
Documentation considerations
Clinical pearls
Case Simulations (during workshop)
Debrief (during workshop)
Questions and answers (during workshop)

## Slide 3
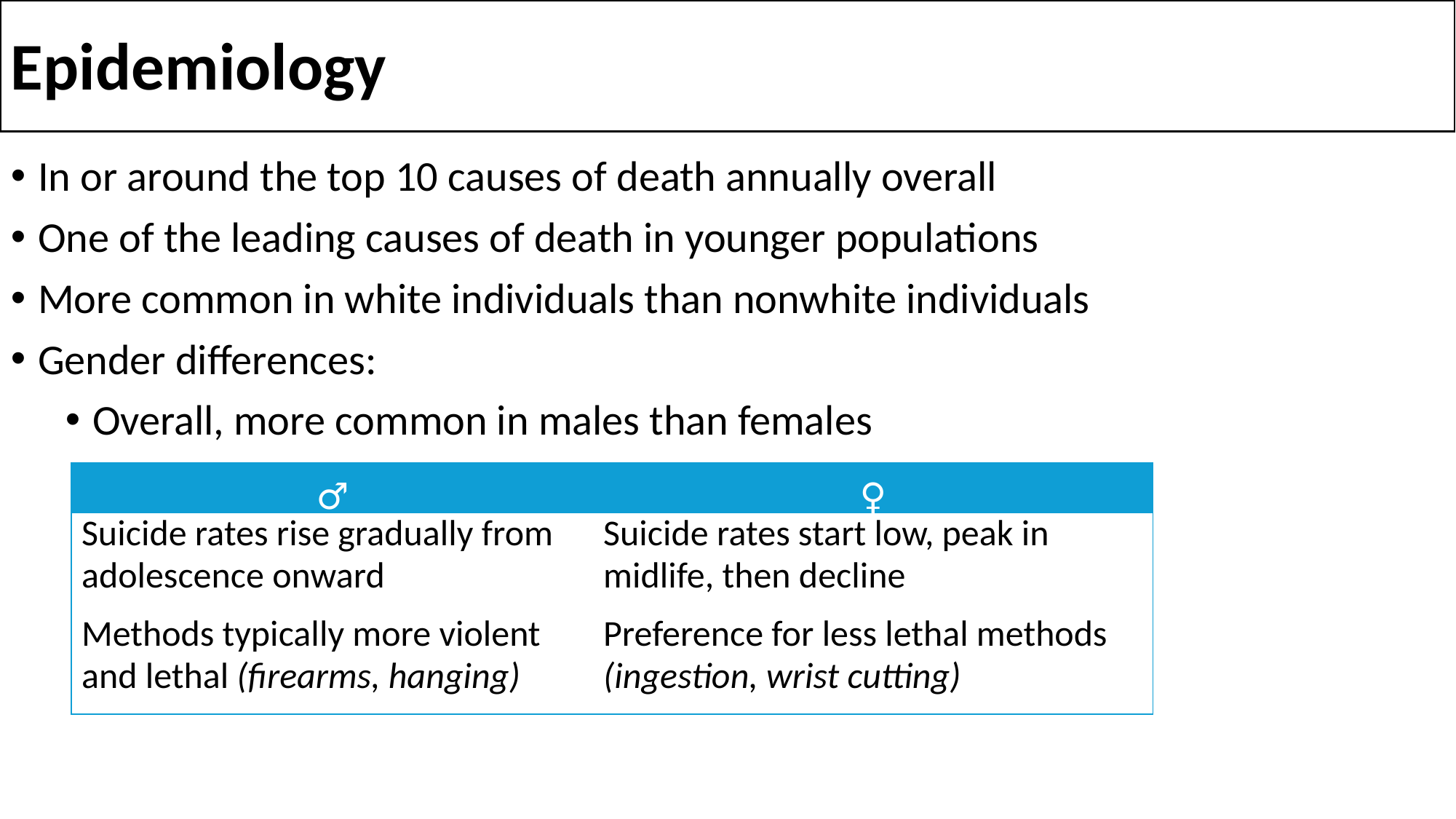

Epidemiology
In or around the top 10 causes of death annually overall
One of the leading causes of death in younger populations
More common in white individuals than nonwhite individuals
Gender differences:
Overall, more common in males than females
| ♂ | ♀ |
| --- | --- |
| Suicide rates rise gradually from adolescence onward | Suicide rates start low, peak in midlife, then decline |
| Methods typically more violent and lethal (firearms, hanging) | Preference for less lethal methods (ingestion, wrist cutting) |

## Slide 4
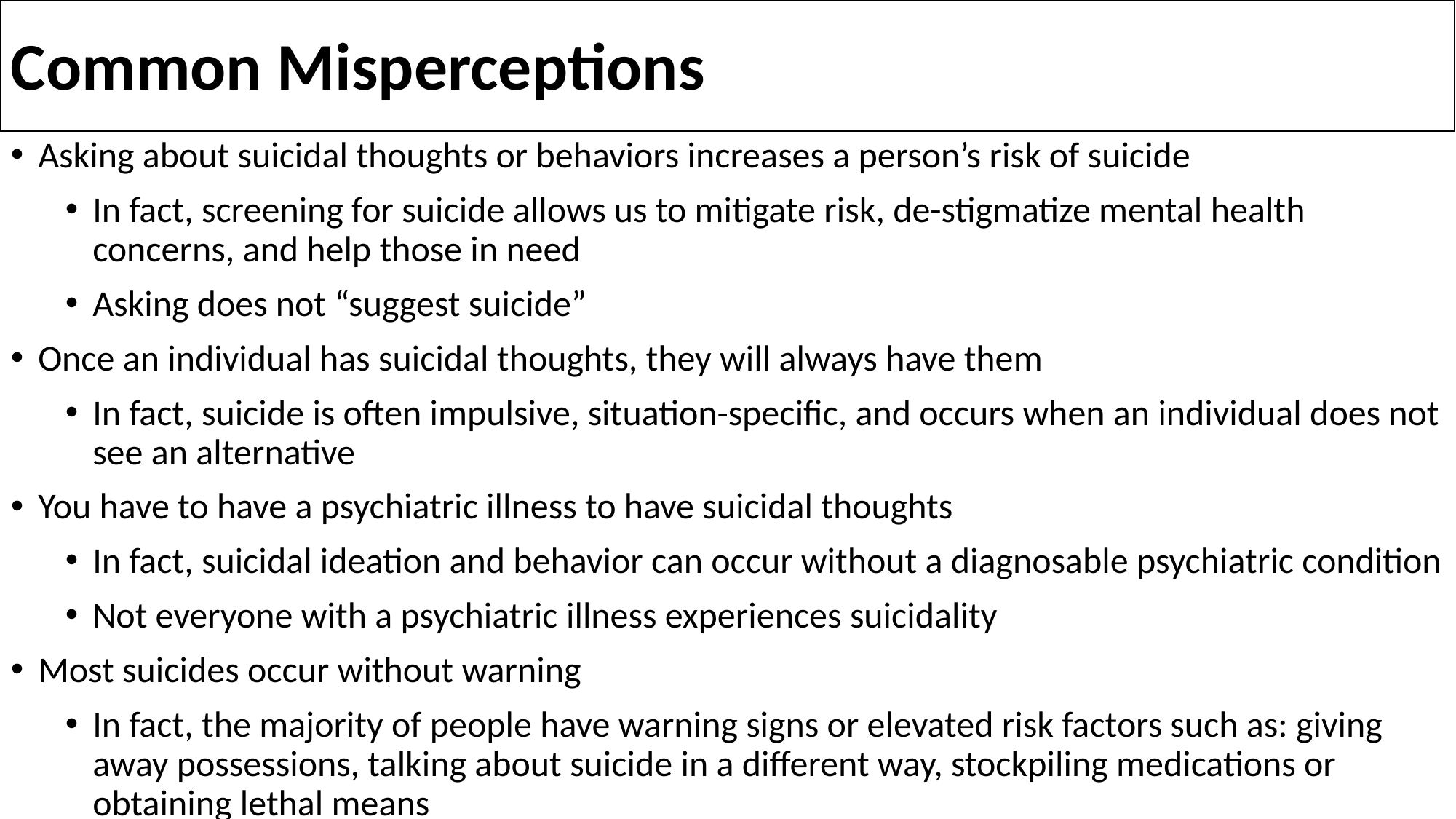

Common Misperceptions
Asking about suicidal thoughts or behaviors increases a person’s risk of suicide
In fact, screening for suicide allows us to mitigate risk, de-stigmatize mental health concerns, and help those in need
Asking does not “suggest suicide”
Once an individual has suicidal thoughts, they will always have them
In fact, suicide is often impulsive, situation-specific, and occurs when an individual does not see an alternative
You have to have a psychiatric illness to have suicidal thoughts
In fact, suicidal ideation and behavior can occur without a diagnosable psychiatric condition
Not everyone with a psychiatric illness experiences suicidality
Most suicides occur without warning
In fact, the majority of people have warning signs or elevated risk factors such as: giving away possessions, talking about suicide in a different way, stockpiling medications or obtaining lethal means

## Slide 5
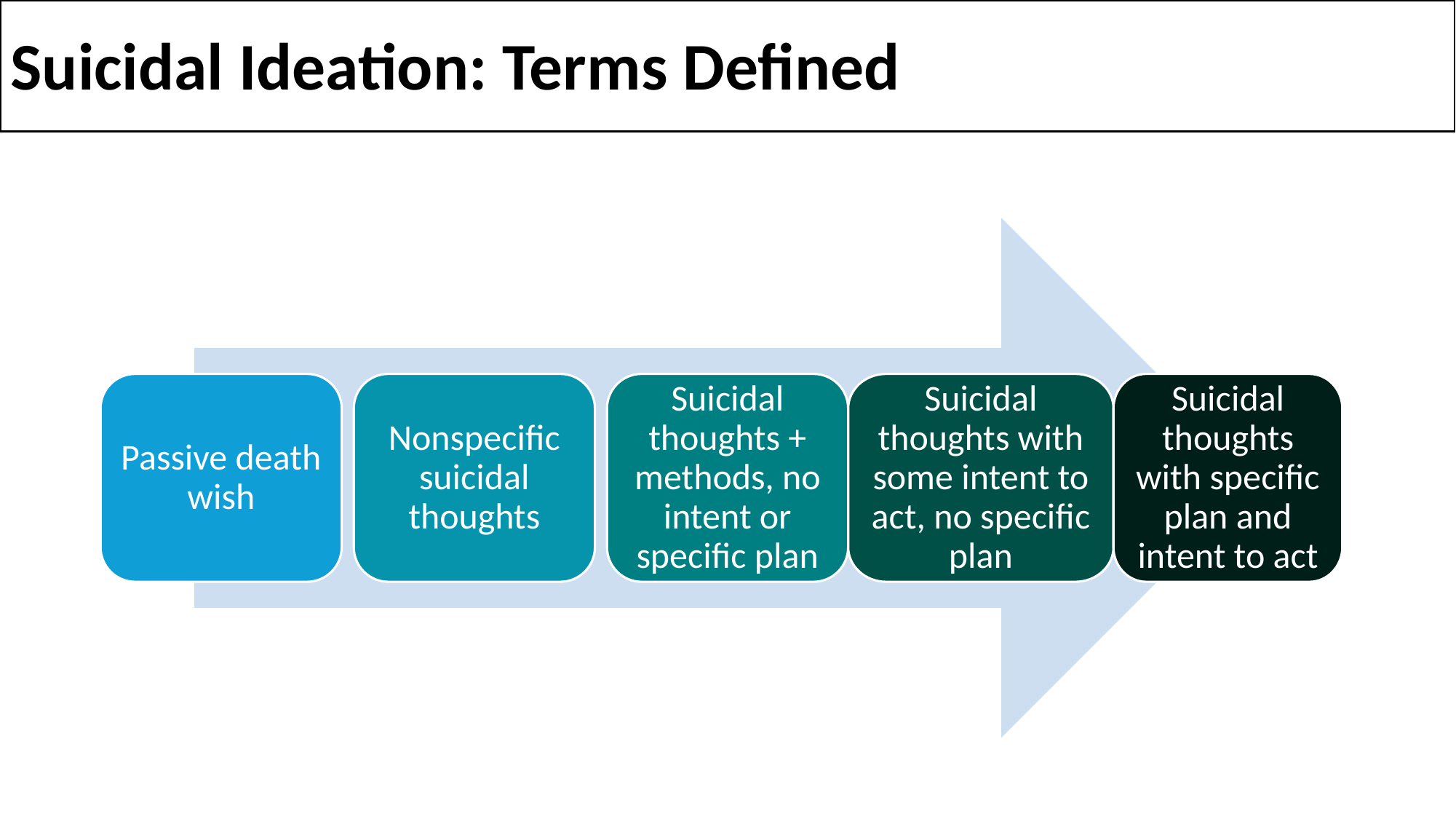

Suicidal Ideation: Terms Defined
Passive death wish
Nonspecific suicidal thoughts
Suicidal thoughts + methods, no intent or specific plan
Suicidal thoughts with some intent to act, no specific plan
Suicidal thoughts with specific plan and intent to act

## Slide 6
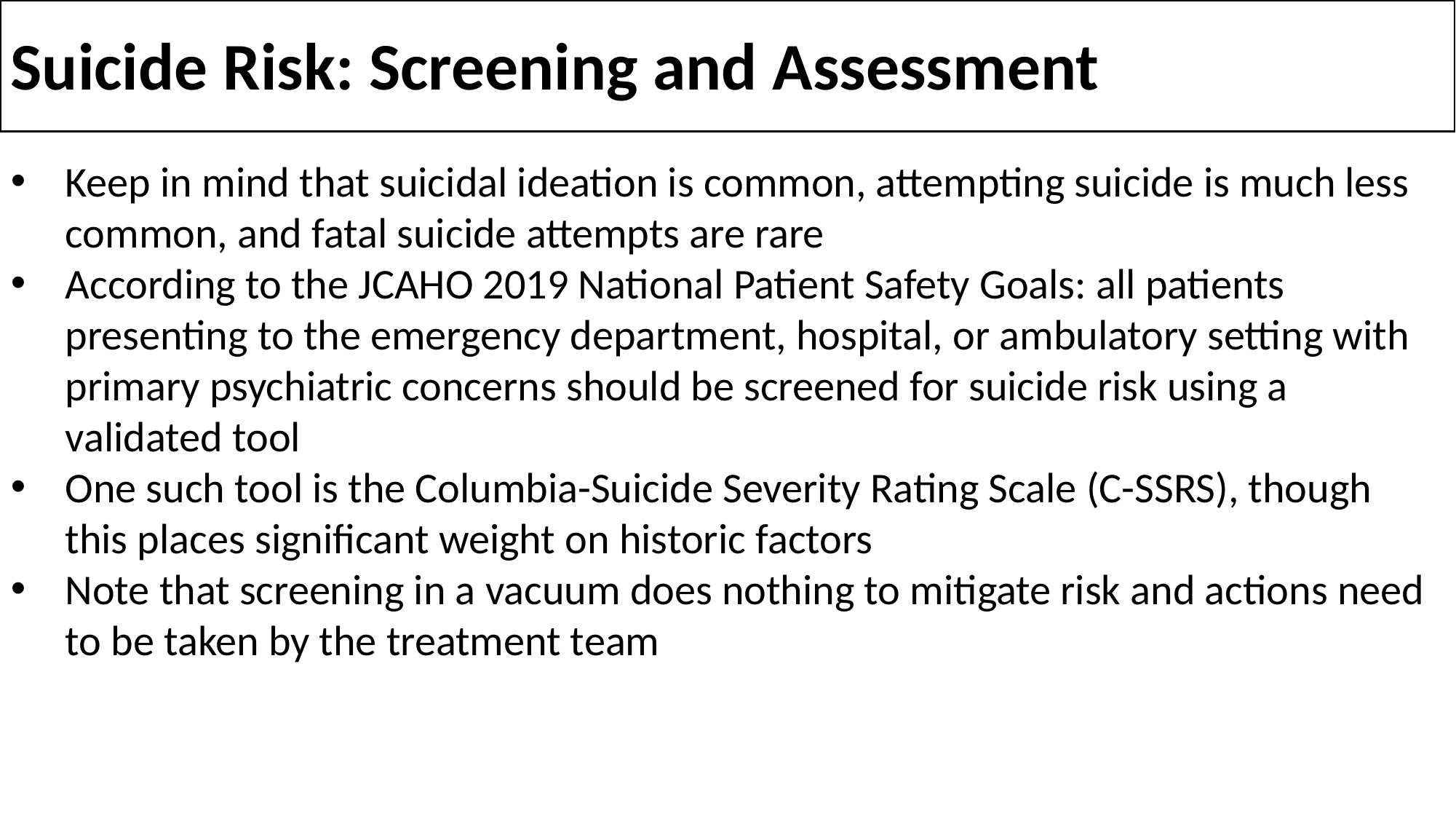

Suicide Risk: Screening and Assessment
Keep in mind that suicidal ideation is common, attempting suicide is much less common, and fatal suicide attempts are rare
According to the JCAHO 2019 National Patient Safety Goals: all patients presenting to the emergency department, hospital, or ambulatory setting with primary psychiatric concerns should be screened for suicide risk using a validated tool
One such tool is the Columbia-Suicide Severity Rating Scale (C-SSRS), though this places significant weight on historic factors
Note that screening in a vacuum does nothing to mitigate risk and actions need to be taken by the treatment team

## Slide 7
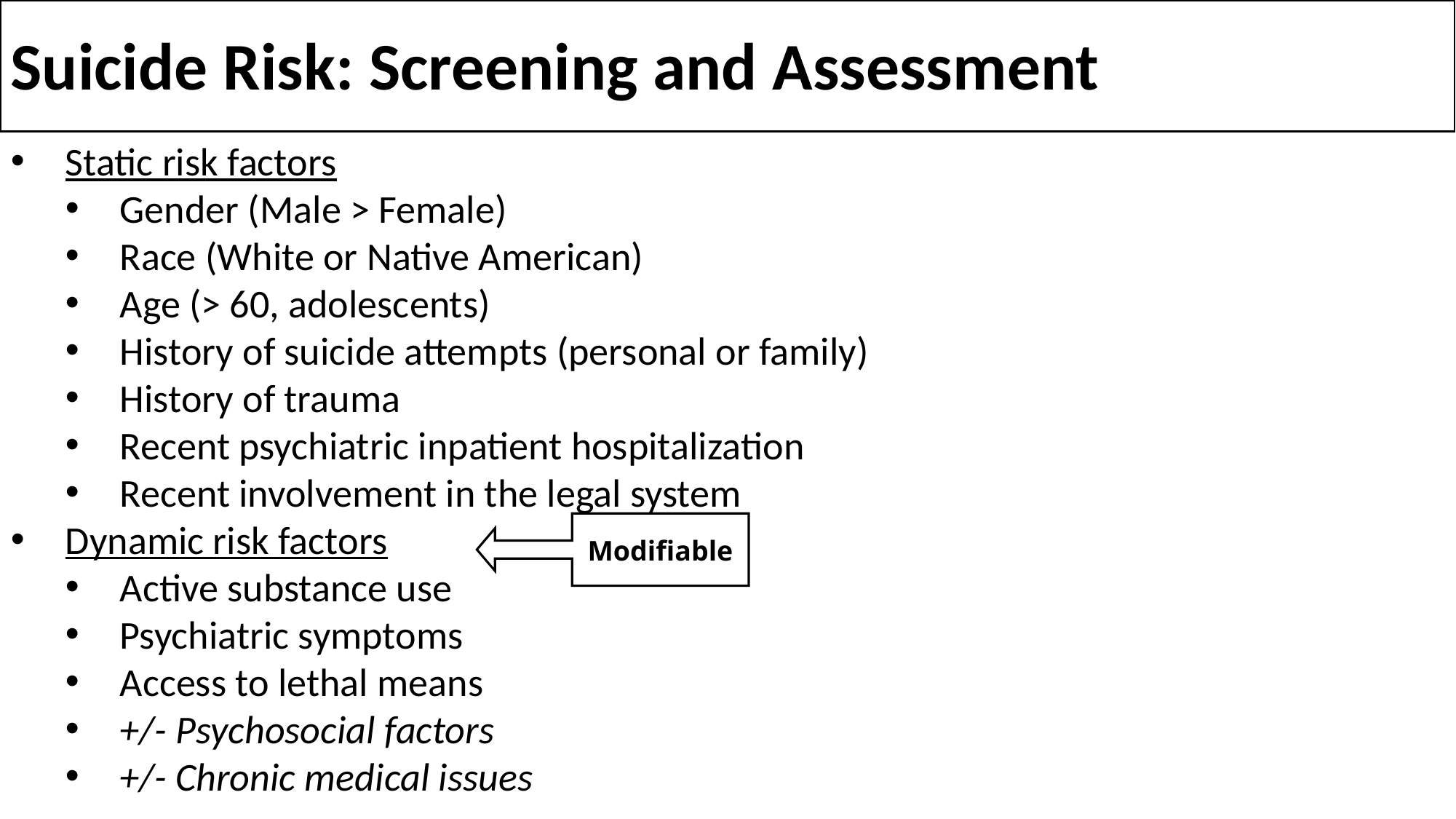

Suicide Risk: Screening and Assessment
Static risk factors
Gender (Male > Female)
Race (White or Native American)
Age (> 60, adolescents)
History of suicide attempts (personal or family)
History of trauma
Recent psychiatric inpatient hospitalization
Recent involvement in the legal system
Dynamic risk factors
Active substance use
Psychiatric symptoms
Access to lethal means
+/- Psychosocial factors
+/- Chronic medical issues
Modifiable

## Slide 8
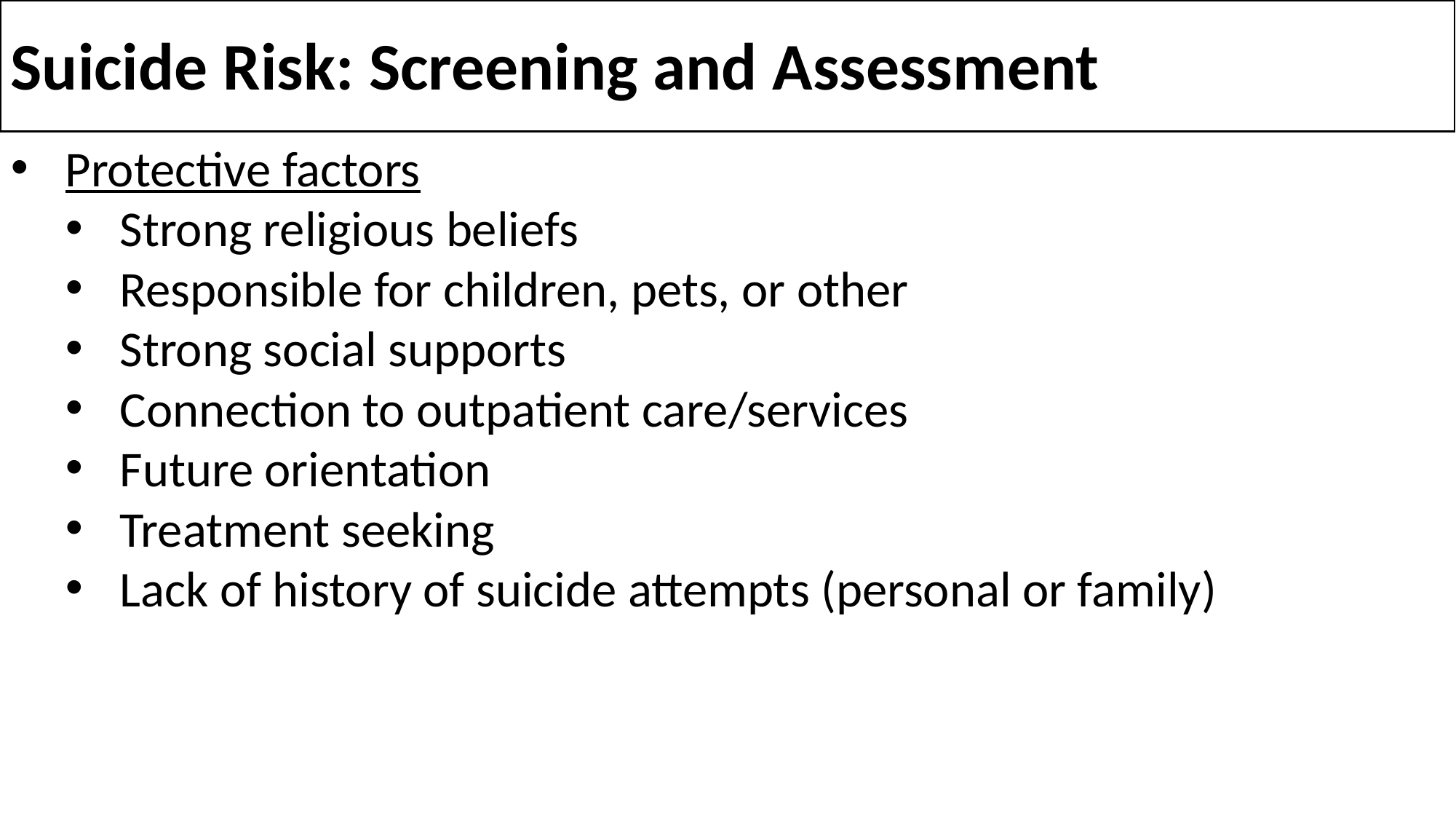

Suicide Risk: Screening and Assessment
Protective factors
Strong religious beliefs
Responsible for children, pets, or other
Strong social supports
Connection to outpatient care/services
Future orientation
Treatment seeking
Lack of history of suicide attempts (personal or family)

## Slide 9
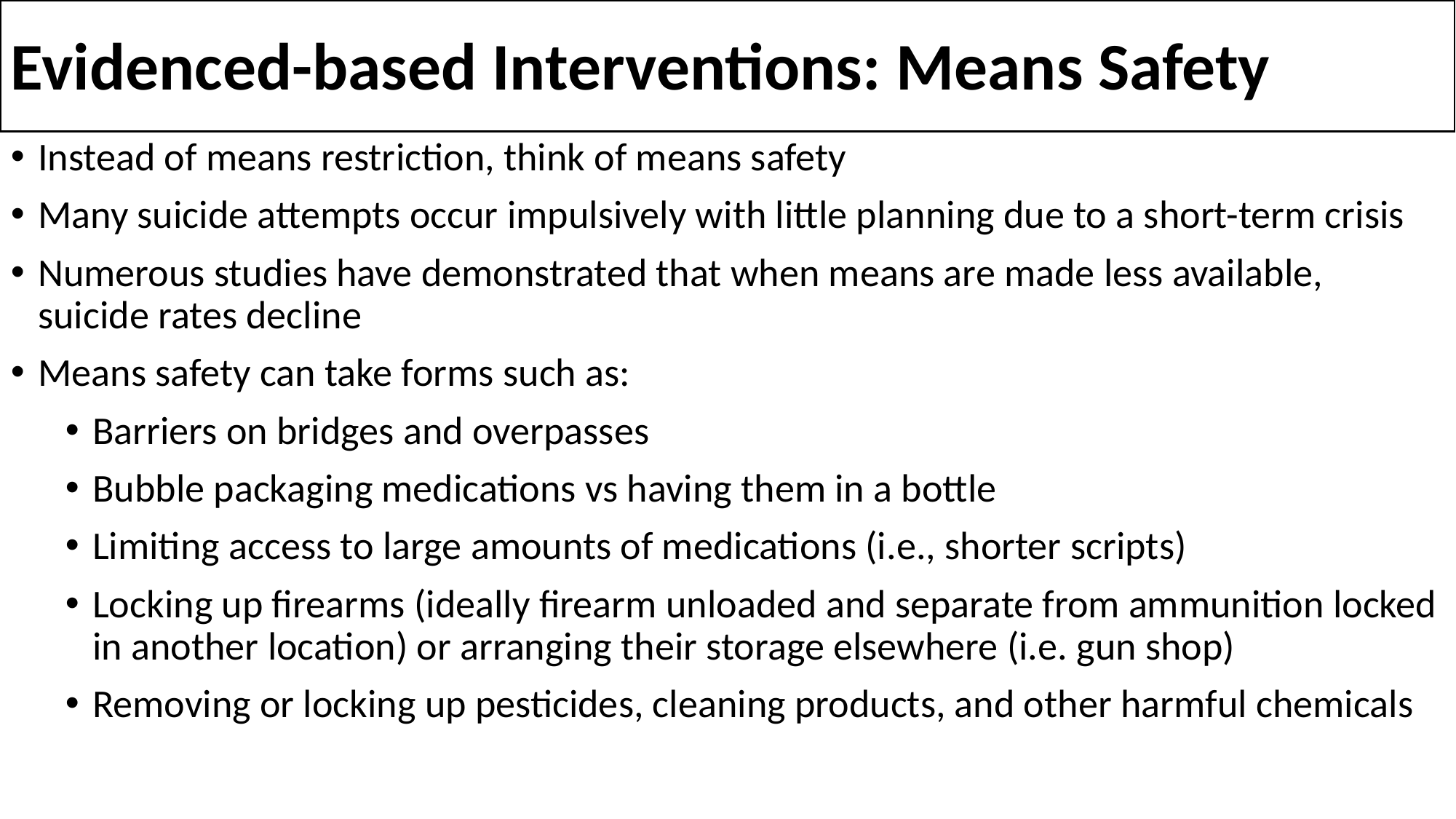

Evidenced-based Interventions: Means Safety
Instead of means restriction, think of means safety
Many suicide attempts occur impulsively with little planning due to a short-term crisis
Numerous studies have demonstrated that when means are made less available, suicide rates decline
Means safety can take forms such as:
Barriers on bridges and overpasses
Bubble packaging medications vs having them in a bottle
Limiting access to large amounts of medications (i.e., shorter scripts)
Locking up firearms (ideally firearm unloaded and separate from ammunition locked in another location) or arranging their storage elsewhere (i.e. gun shop)
Removing or locking up pesticides, cleaning products, and other harmful chemicals

## Slide 10
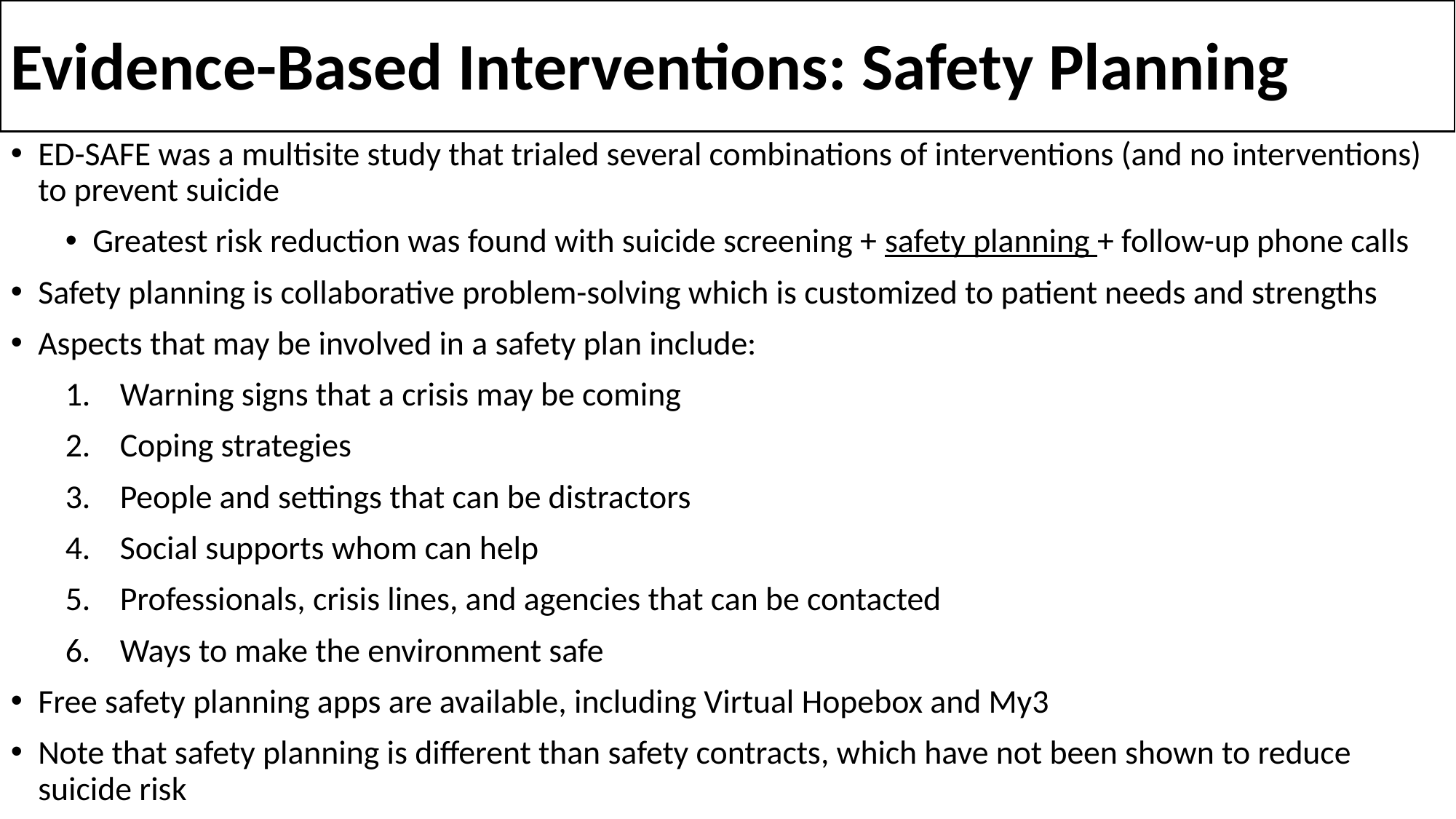

Evidence-Based Interventions: Safety Planning
ED-SAFE was a multisite study that trialed several combinations of interventions (and no interventions) to prevent suicide
Greatest risk reduction was found with suicide screening + safety planning + follow-up phone calls
Safety planning is collaborative problem-solving which is customized to patient needs and strengths
Aspects that may be involved in a safety plan include:
Warning signs that a crisis may be coming
Coping strategies
People and settings that can be distractors
Social supports whom can help
Professionals, crisis lines, and agencies that can be contacted
Ways to make the environment safe
Free safety planning apps are available, including Virtual Hopebox and My3
Note that safety planning is different than safety contracts, which have not been shown to reduce suicide risk

## Slide 11
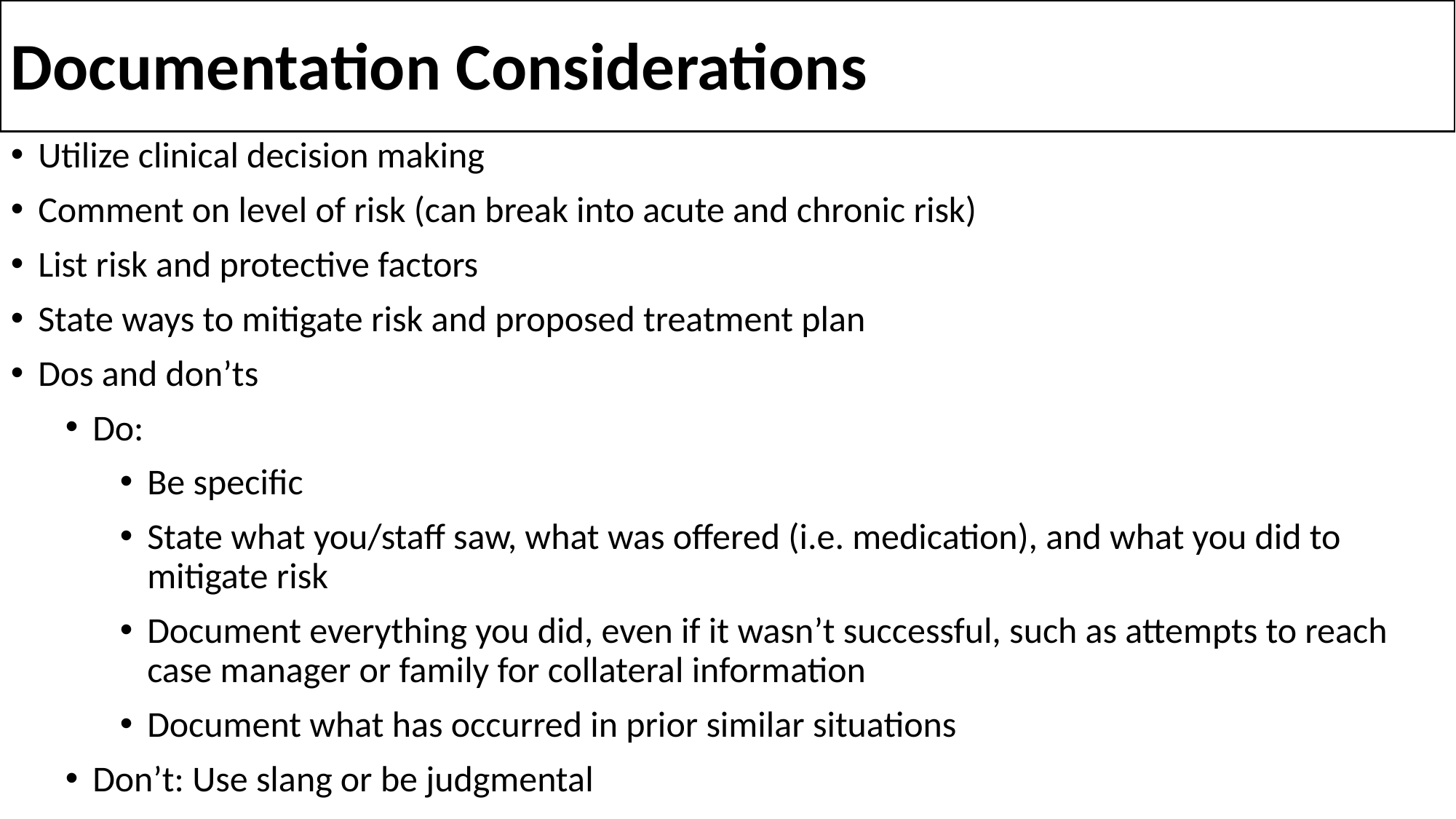

Documentation Considerations
Utilize clinical decision making
Comment on level of risk (can break into acute and chronic risk)
List risk and protective factors
State ways to mitigate risk and proposed treatment plan
Dos and don’ts
Do:
Be specific
State what you/staff saw, what was offered (i.e. medication), and what you did to mitigate risk
Document everything you did, even if it wasn’t successful, such as attempts to reach case manager or family for collateral information
Document what has occurred in prior similar situations
Don’t: Use slang or be judgmental

## Slide 12
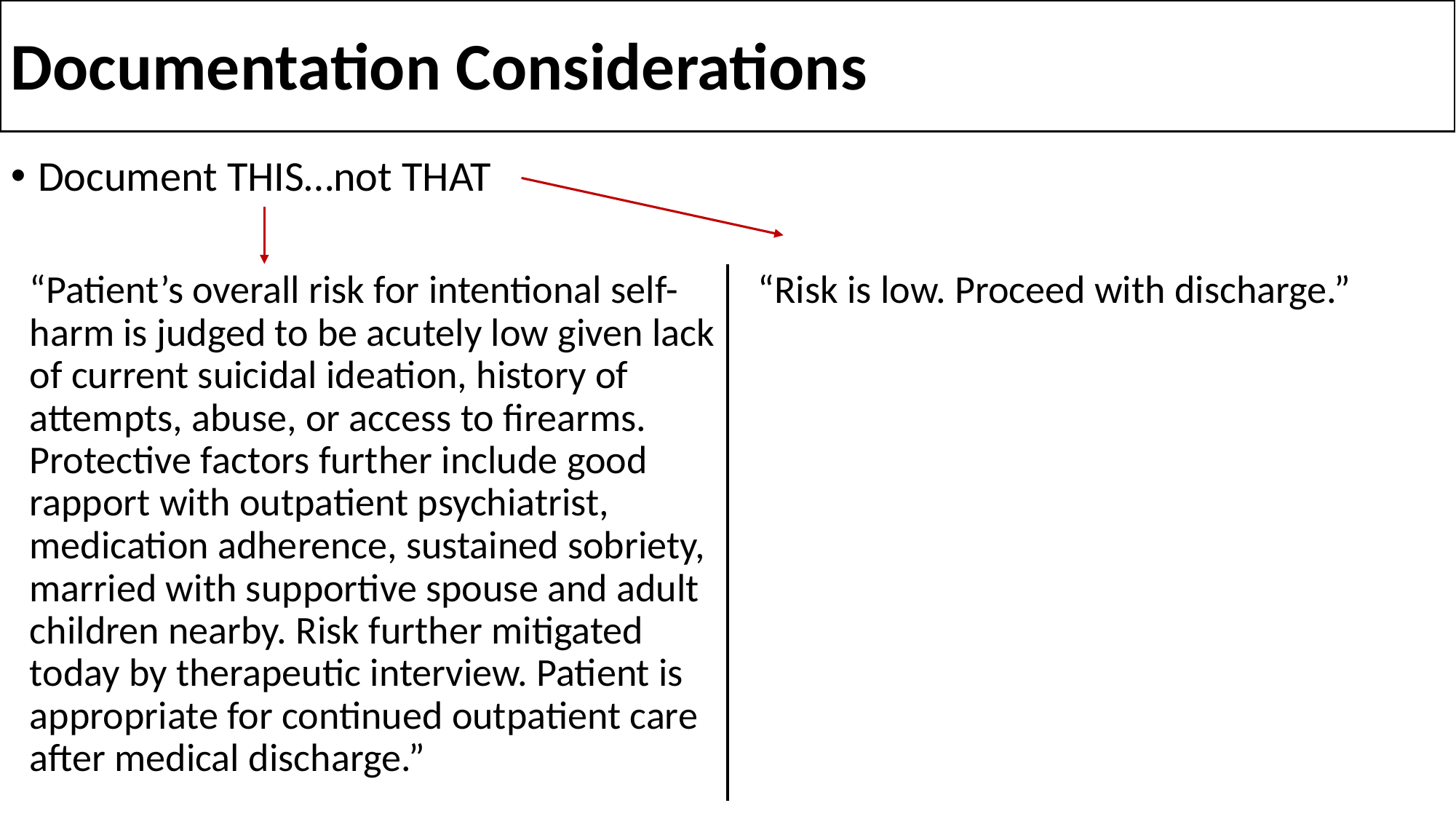

Documentation Considerations
Document THIS…not THAT
“Patient’s overall risk for intentional self-harm is judged to be acutely low given lack of current suicidal ideation, history of attempts, abuse, or access to firearms. Protective factors further include good rapport with outpatient psychiatrist, medication adherence, sustained sobriety, married with supportive spouse and adult children nearby. Risk further mitigated today by therapeutic interview. Patient is appropriate for continued outpatient care after medical discharge.”
“Risk is low. Proceed with discharge.”

## Slide 13
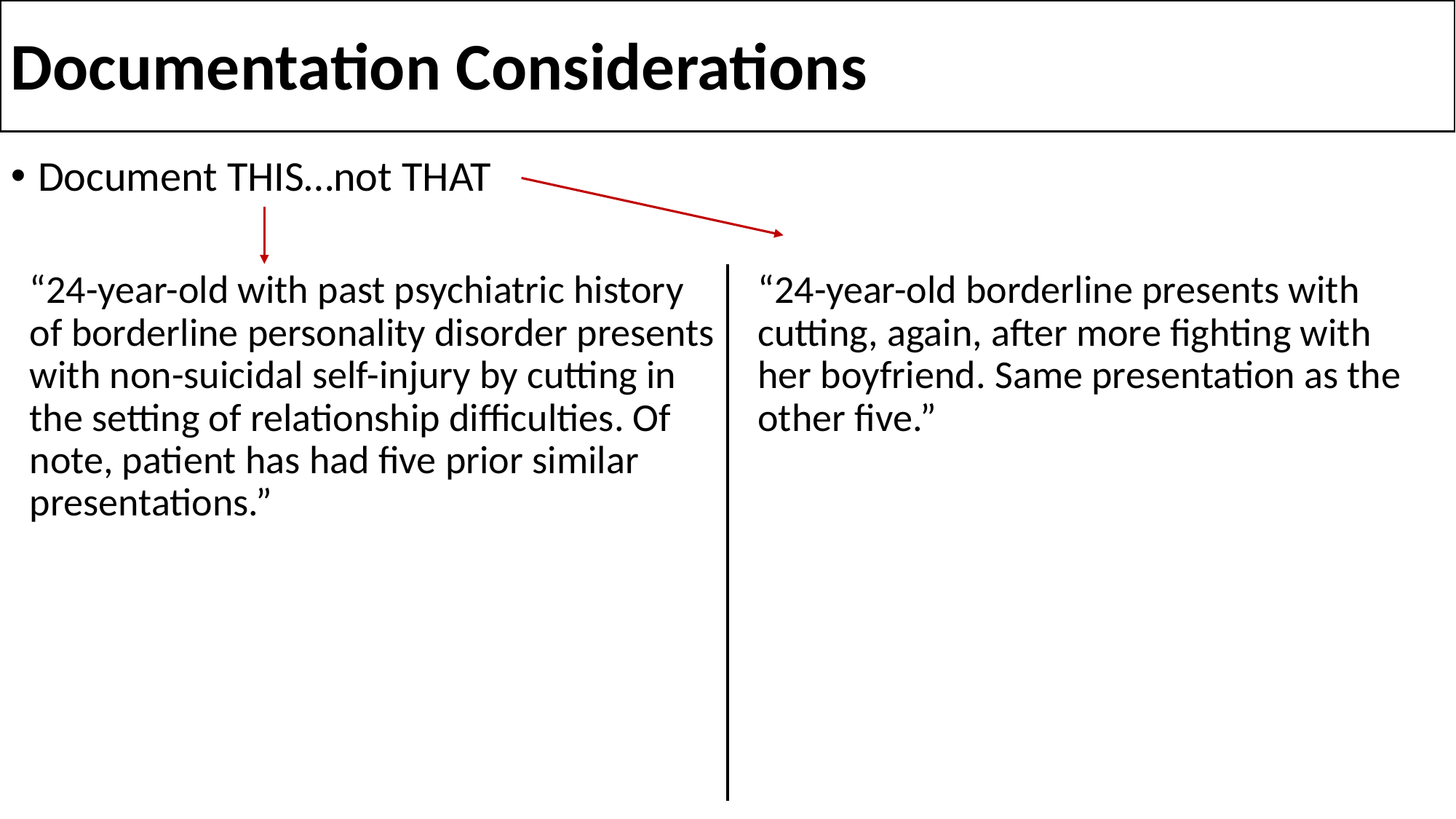

Documentation Considerations
Document THIS…not THAT
“24-year-old with past psychiatric history of borderline personality disorder presents with non-suicidal self-injury by cutting in the setting of relationship difficulties. Of note, patient has had five prior similar presentations.”
“24-year-old borderline presents with cutting, again, after more fighting with her boyfriend. Same presentation as the other five.”

## Slide 14
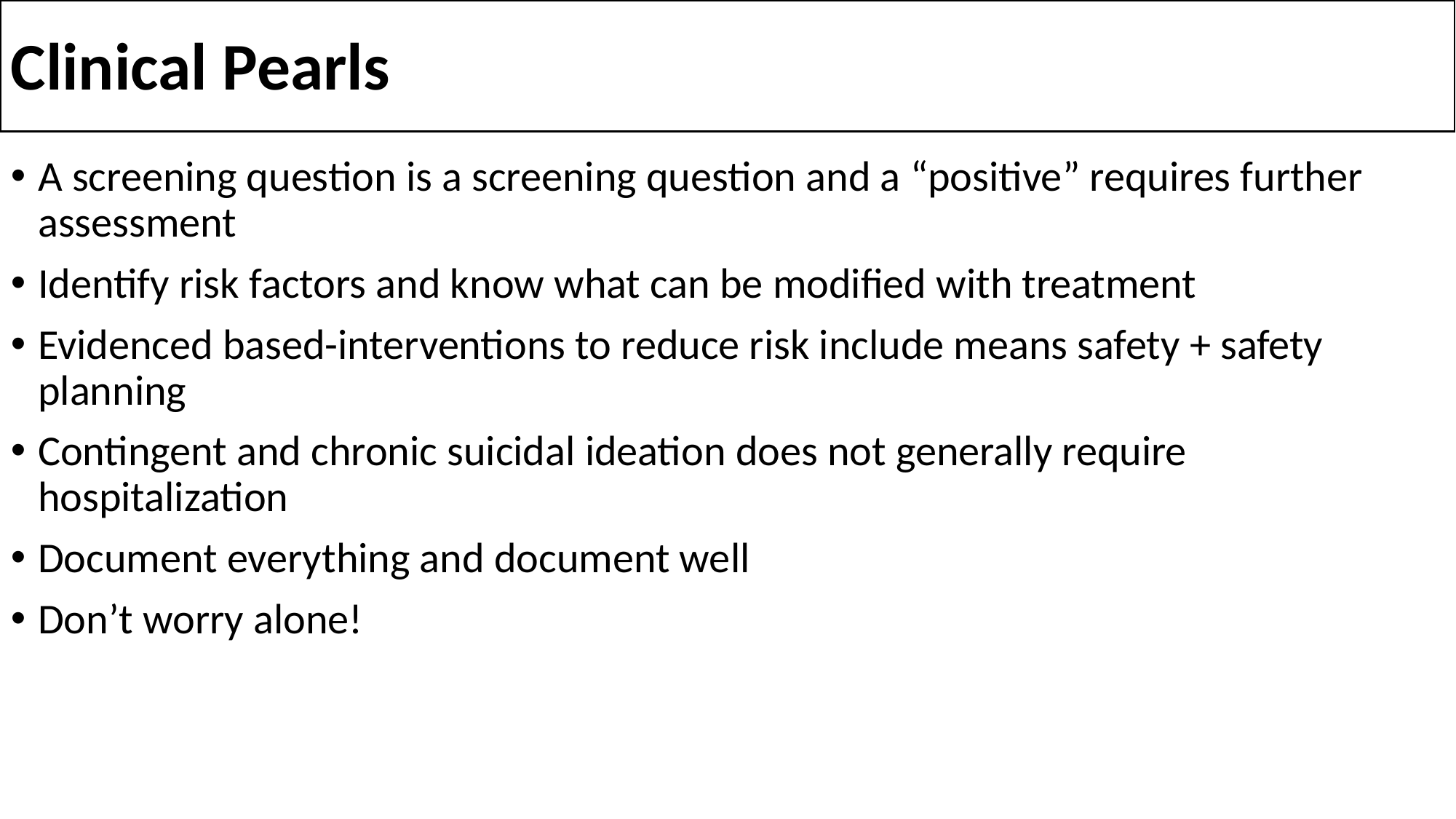

Clinical Pearls
A screening question is a screening question and a “positive” requires further assessment
Identify risk factors and know what can be modified with treatment
Evidenced based-interventions to reduce risk include means safety + safety planning
Contingent and chronic suicidal ideation does not generally require hospitalization
Document everything and document well
Don’t worry alone!

## Slide 15
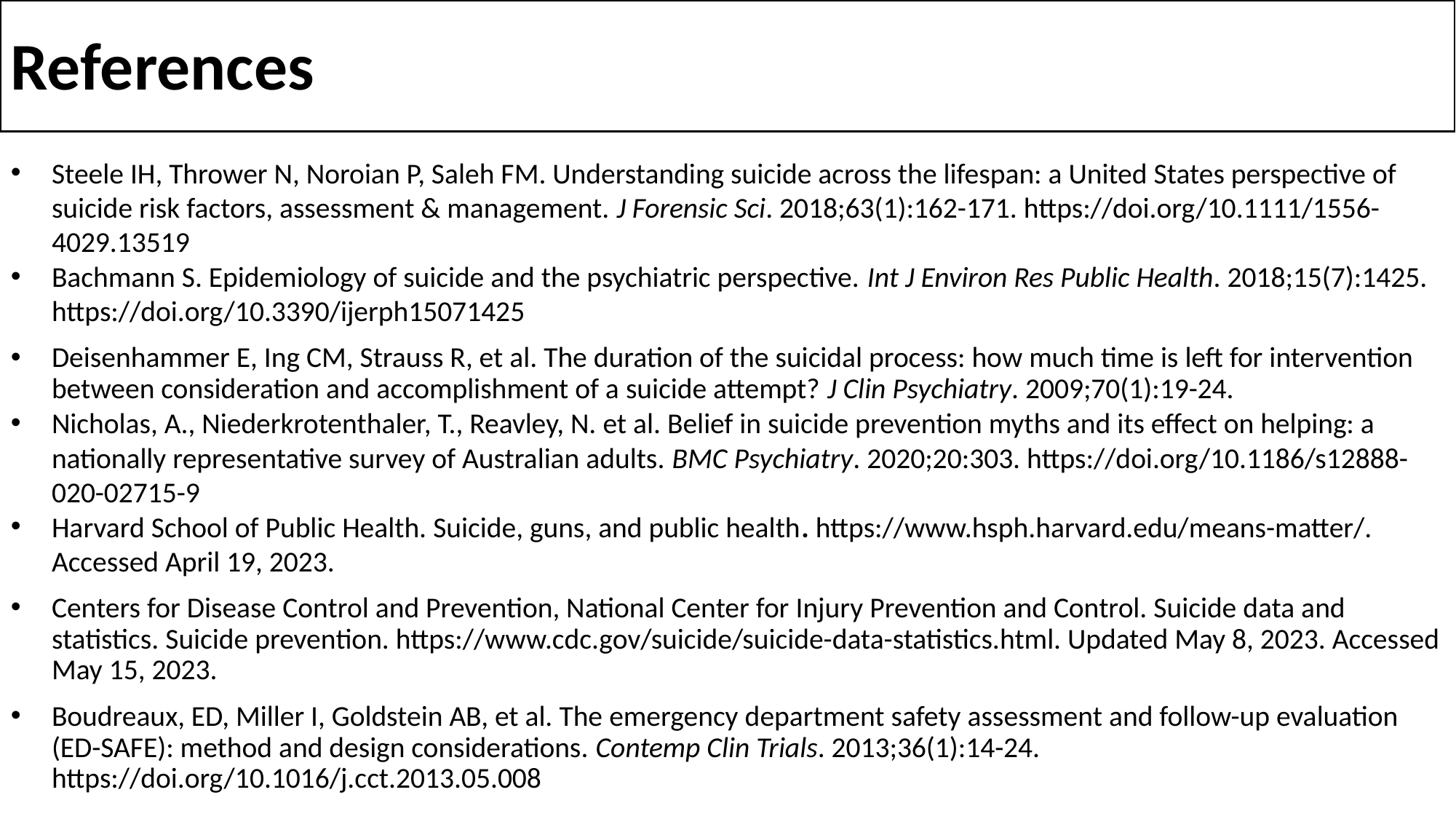

References
Steele IH, Thrower N, Noroian P, Saleh FM. Understanding suicide across the lifespan: a United States perspective of suicide risk factors, assessment & management. J Forensic Sci. 2018;63(1):162-171. https://doi.org/10.1111/1556-4029.13519
Bachmann S. Epidemiology of suicide and the psychiatric perspective. Int J Environ Res Public Health. 2018;15(7):1425. https://doi.org/10.3390/ijerph15071425
Deisenhammer E, Ing CM, Strauss R, et al. The duration of the suicidal process: how much time is left for intervention between consideration and accomplishment of a suicide attempt? J Clin Psychiatry. 2009;70(1):19-24.
Nicholas, A., Niederkrotenthaler, T., Reavley, N. et al. Belief in suicide prevention myths and its effect on helping: a nationally representative survey of Australian adults. BMC Psychiatry. 2020;20:303. https://doi.org/10.1186/s12888-020-02715-9
Harvard School of Public Health. Suicide, guns, and public health. https://www.hsph.harvard.edu/means-matter/. Accessed April 19, 2023.
Centers for Disease Control and Prevention, National Center for Injury Prevention and Control. Suicide data and statistics. Suicide prevention. https://www.cdc.gov/suicide/suicide-data-statistics.html. Updated May 8, 2023. Accessed May 15, 2023.
Boudreaux, ED, Miller I, Goldstein AB, et al. The emergency department safety assessment and follow-up evaluation (ED-SAFE): method and design considerations. Contemp Clin Trials. 2013;36(1):14-24. https://doi.org/10.1016/j.cct.2013.05.008
