# Supplementary material for: A Multimodal Workshop to Improve Medical Student Self-Assessment of Knowledge and Comfort Managing Patients With Suicidality
Source: MedEdPORTAL. 2025 Jan 17;21:11488. doi: 10.15766/mep_2374-8265.11488 (PMC11739282; doi:10.15766/mep_2374-8265.11488)
Supplement: Supplementary file 1 — SP Case - Joe Jones.docxSP Case - Susan Olson.docxPreworkshop Slides.pptxDidactic and Group Discussion Slides.pptxCase of Joe Jones Door Card.docxCase of Susan Olson Door Card.docxSP encounter Facilitator Guide.docxPreworkshop Survey.docxPostworkshop Survey.docx [file mep_2374-8265.11488-s001.zip › D. Didactic and Group Discussion Slides.pptx]

## Slide 1
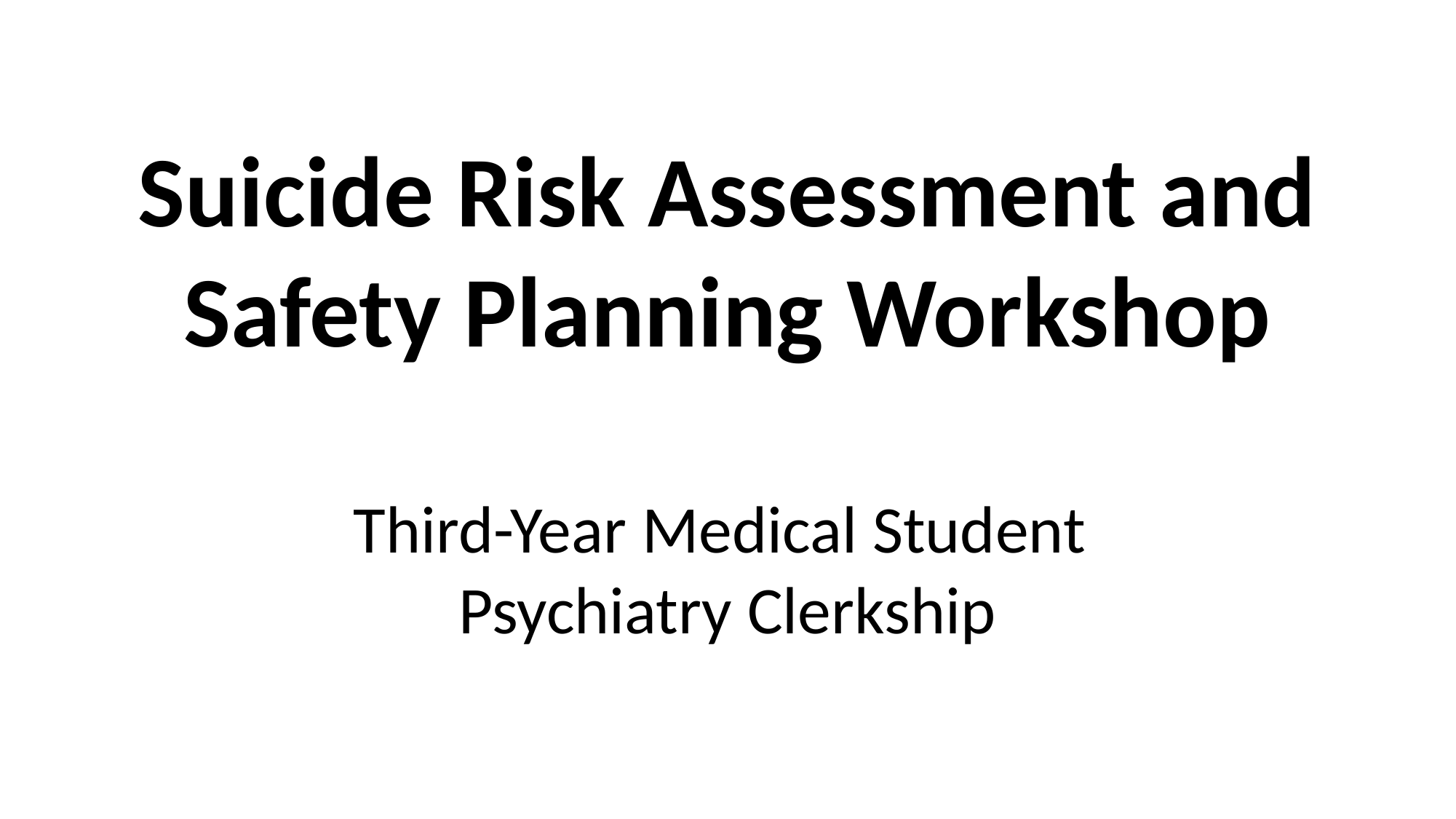

Suicide Risk Assessment and Safety Planning Workshop
Third-Year Medical Student
Psychiatry Clerkship

## Slide 2
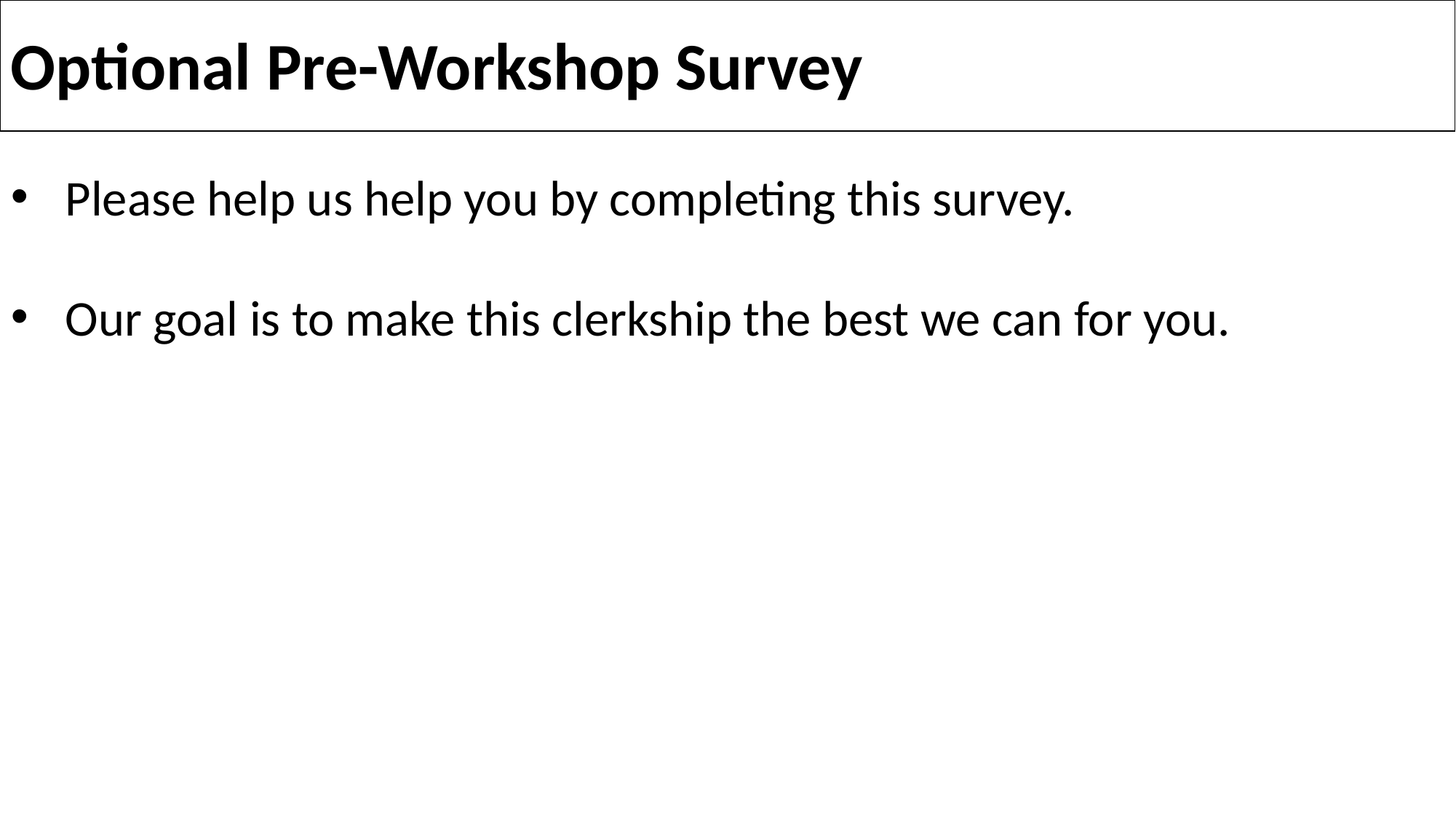

Optional Pre-Workshop Survey
Please help us help you by completing this survey.
Our goal is to make this clerkship the best we can for you.

## Slide 3
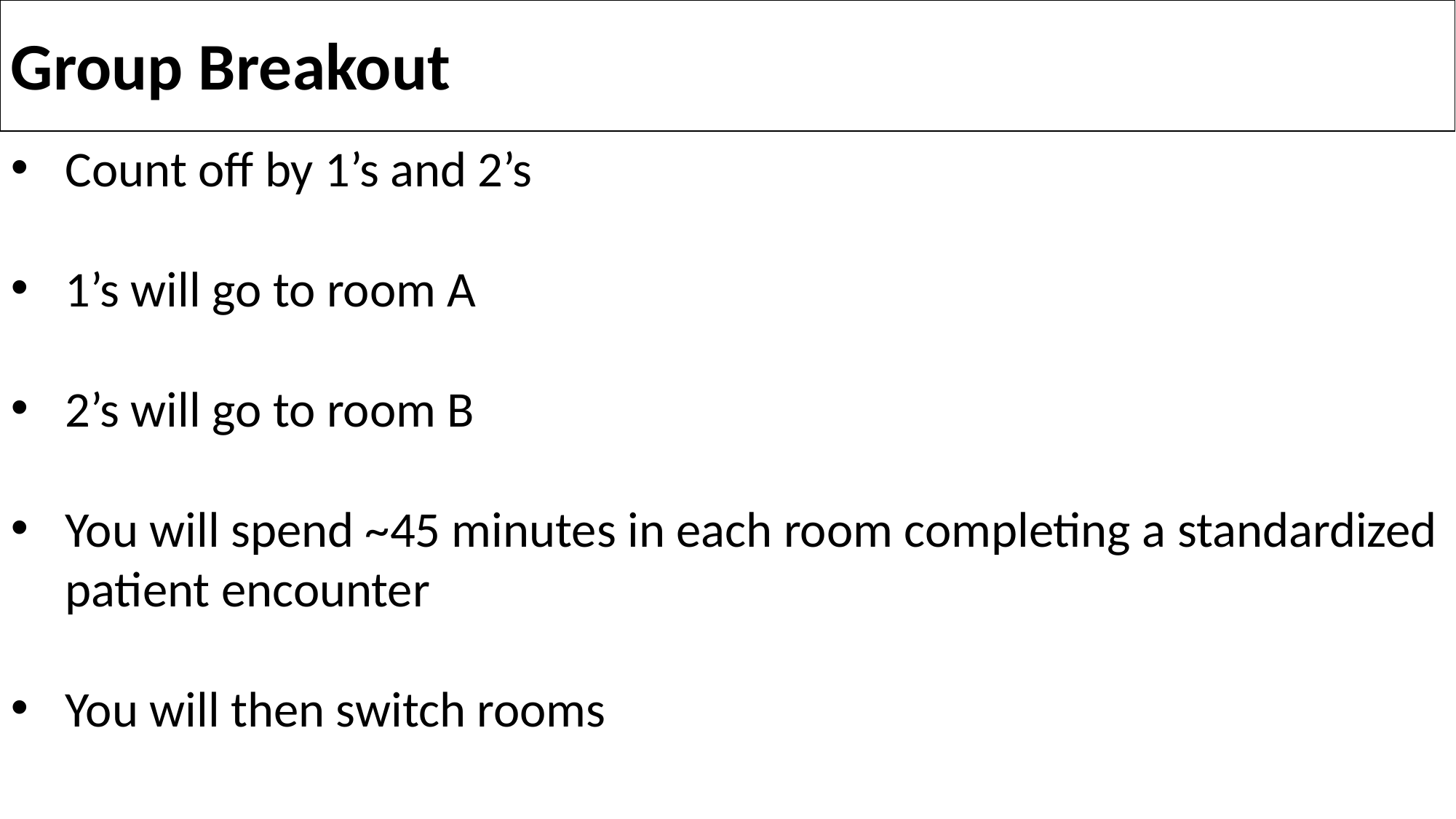

Group Breakout
Count off by 1’s and 2’s
1’s will go to room A
2’s will go to room B
You will spend ~45 minutes in each room completing a standardized patient encounter
You will then switch rooms

## Slide 4
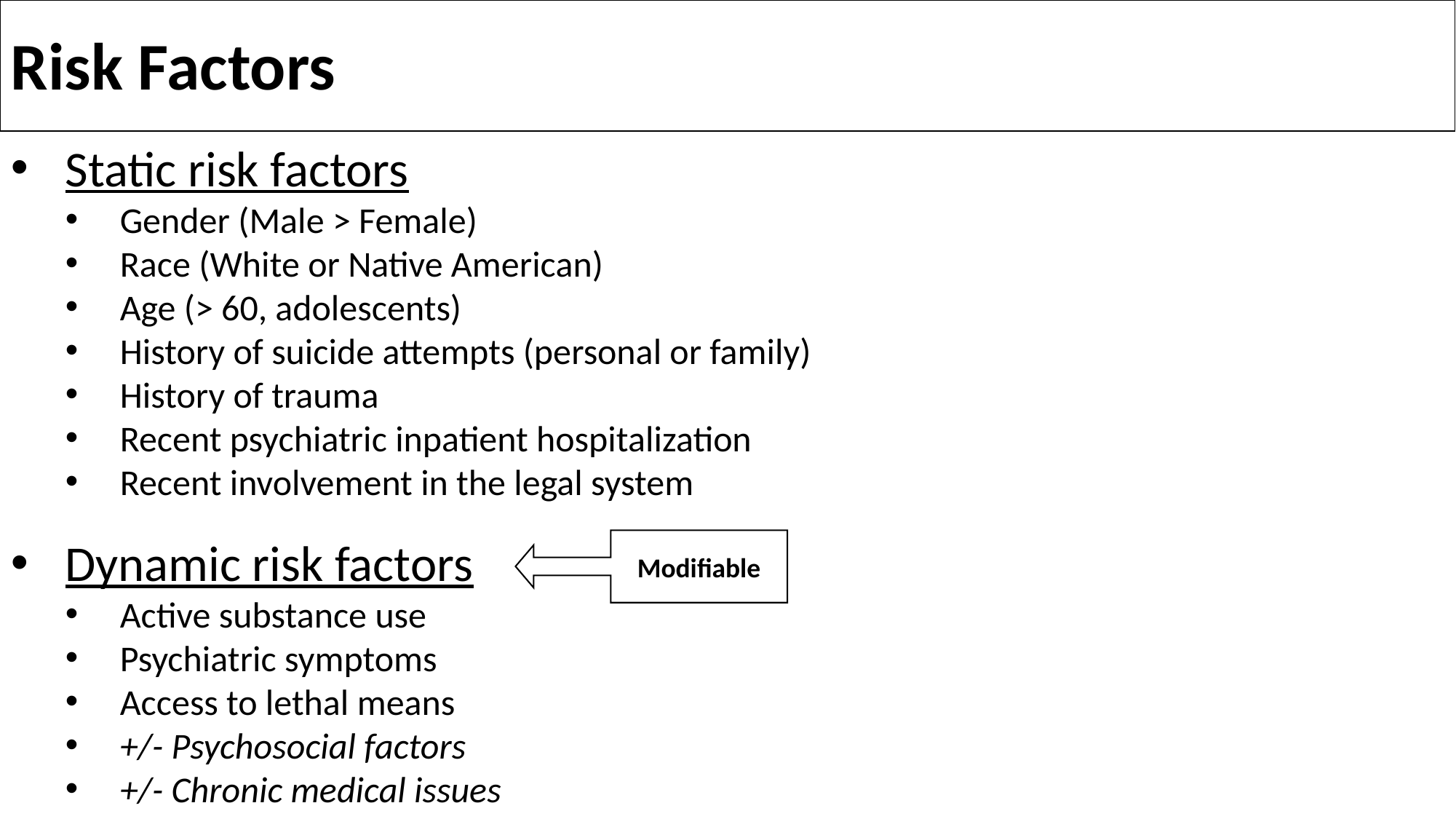

Risk Factors
Static risk factors
Gender (Male > Female)
Race (White or Native American)
Age (> 60, adolescents)
History of suicide attempts (personal or family)
History of trauma
Recent psychiatric inpatient hospitalization
Recent involvement in the legal system
Dynamic risk factors
Active substance use
Psychiatric symptoms
Access to lethal means
+/- Psychosocial factors
+/- Chronic medical issues
Modifiable

## Slide 5
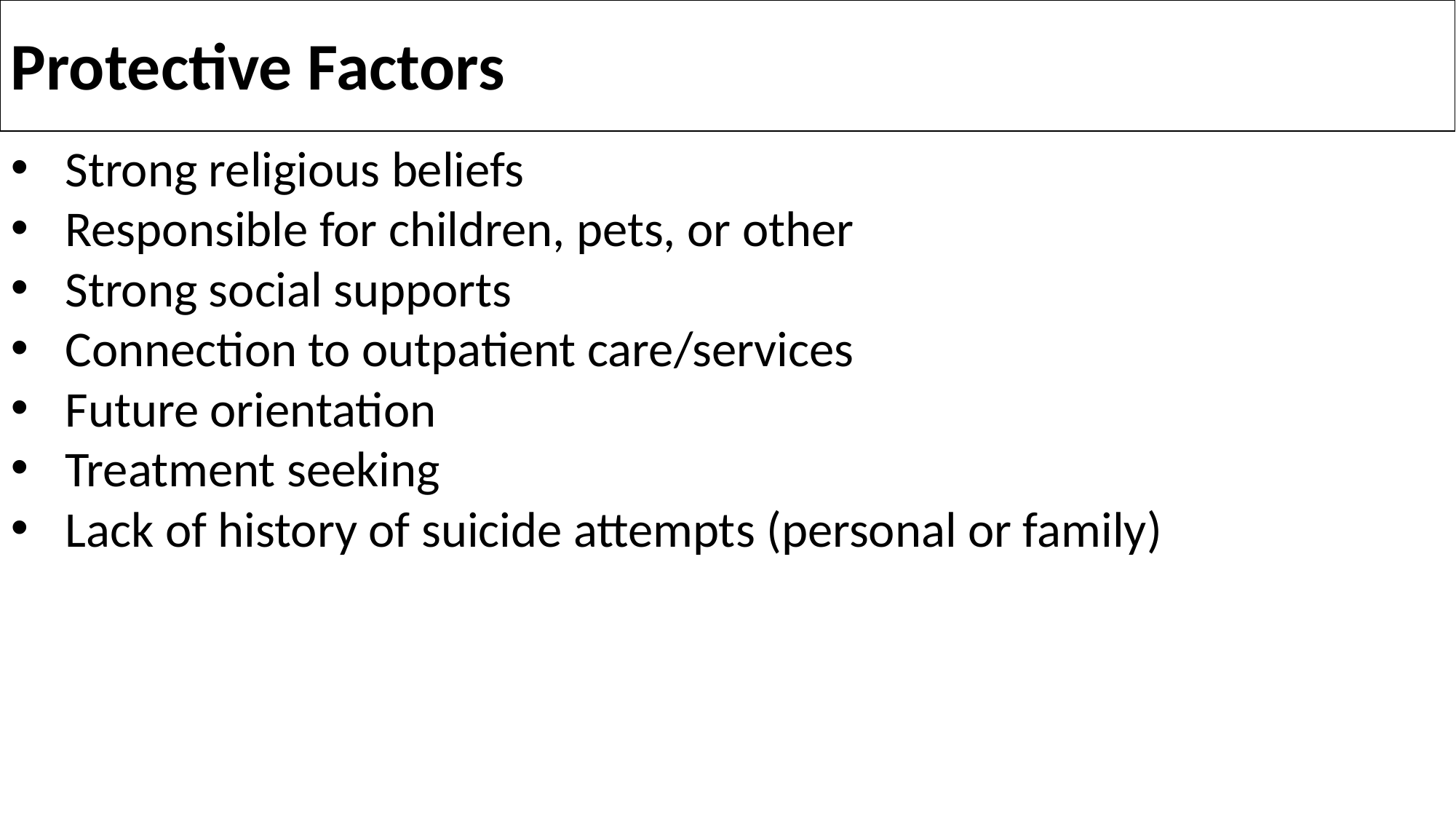

Protective Factors
Strong religious beliefs
Responsible for children, pets, or other
Strong social supports
Connection to outpatient care/services
Future orientation
Treatment seeking
Lack of history of suicide attempts (personal or family)

## Slide 6
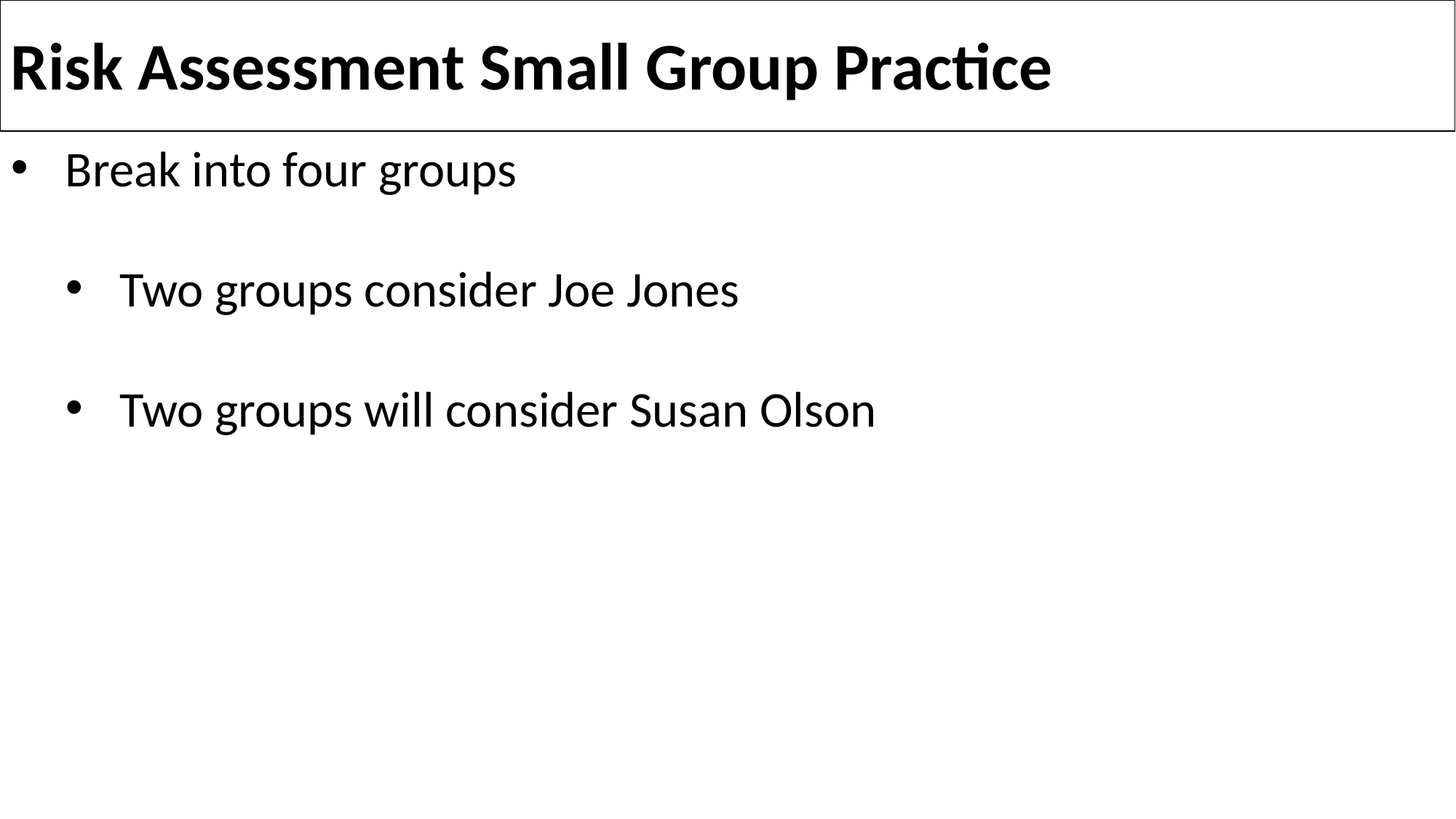

Risk Assessment Small Group Practice
Break into four groups
Two groups consider Joe Jones
Two groups will consider Susan Olson

## Slide 7
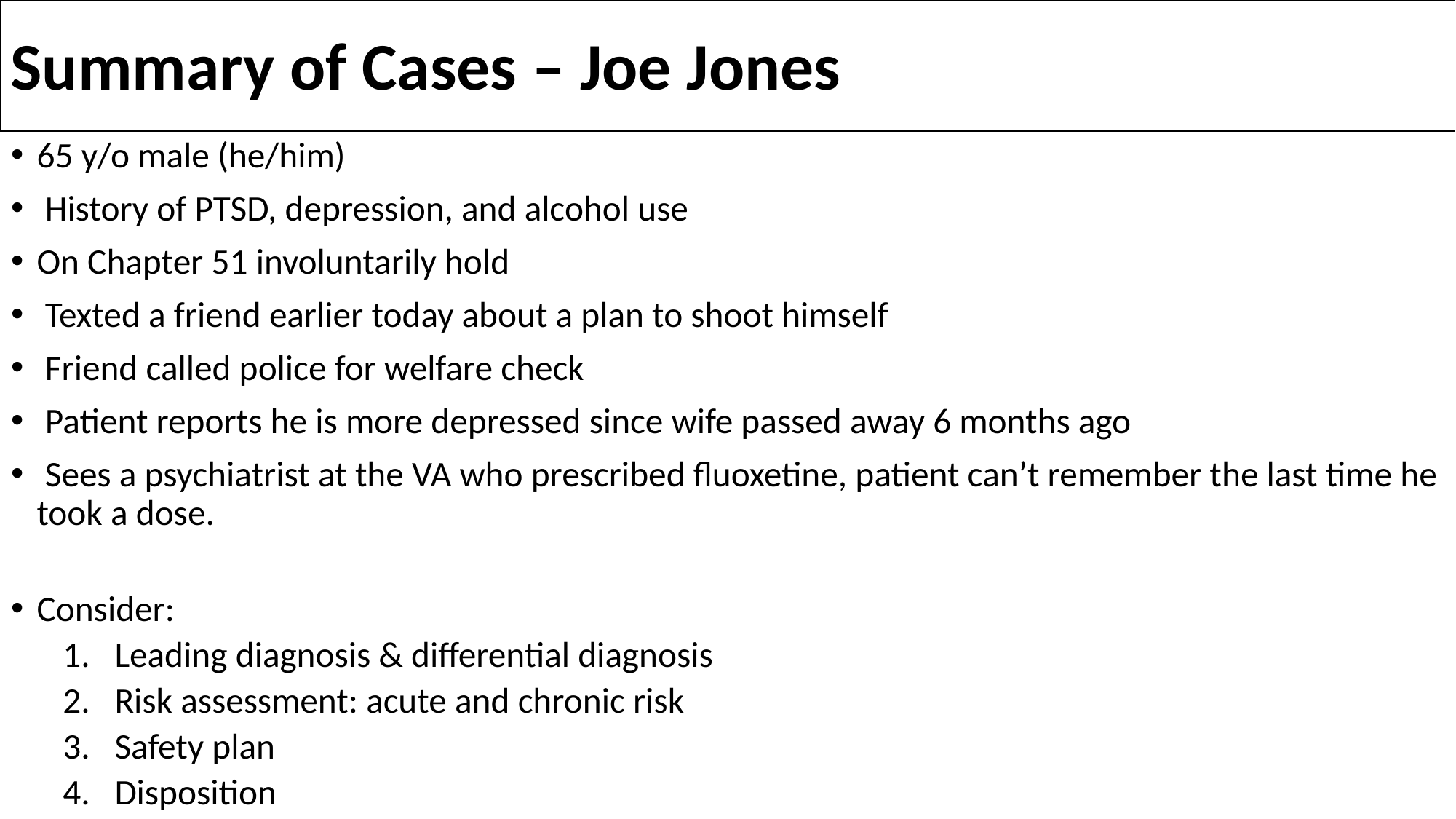

Summary of Cases – Joe Jones
65 y/o male (he/him)
 History of PTSD, depression, and alcohol use
On Chapter 51 involuntarily hold
 Texted a friend earlier today about a plan to shoot himself
 Friend called police for welfare check
 Patient reports he is more depressed since wife passed away 6 months ago
 Sees a psychiatrist at the VA who prescribed fluoxetine, patient can’t remember the last time he took a dose.
Consider:
Leading diagnosis & differential diagnosis
Risk assessment: acute and chronic risk
Safety plan
Disposition

## Slide 8
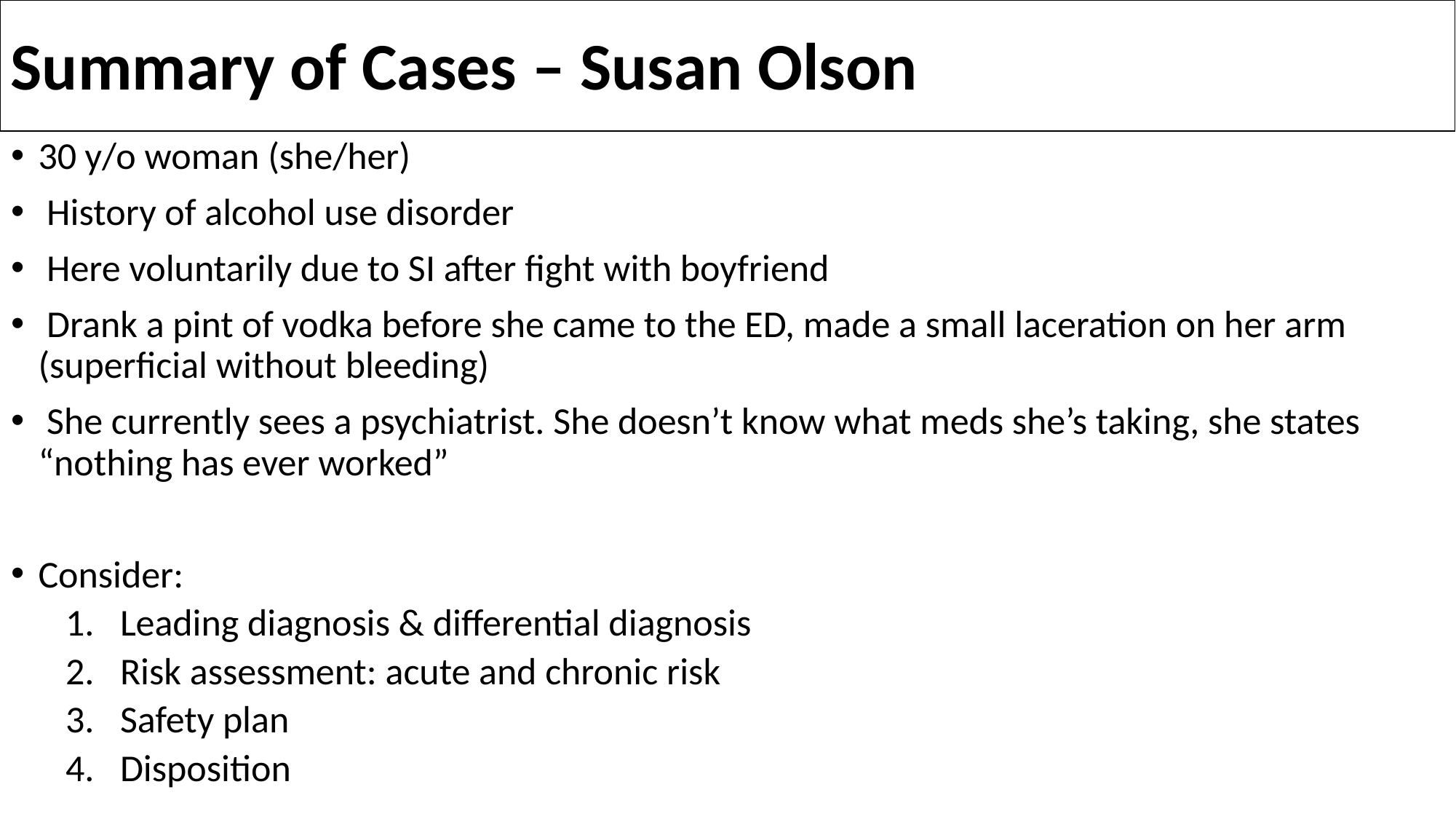

Summary of Cases – Susan Olson
30 y/o woman (she/her)
 History of alcohol use disorder
 Here voluntarily due to SI after fight with boyfriend
 Drank a pint of vodka before she came to the ED, made a small laceration on her arm (superficial without bleeding)
 She currently sees a psychiatrist. She doesn’t know what meds she’s taking, she states “nothing has ever worked”
Consider:
Leading diagnosis & differential diagnosis
Risk assessment: acute and chronic risk
Safety plan
Disposition

## Slide 9
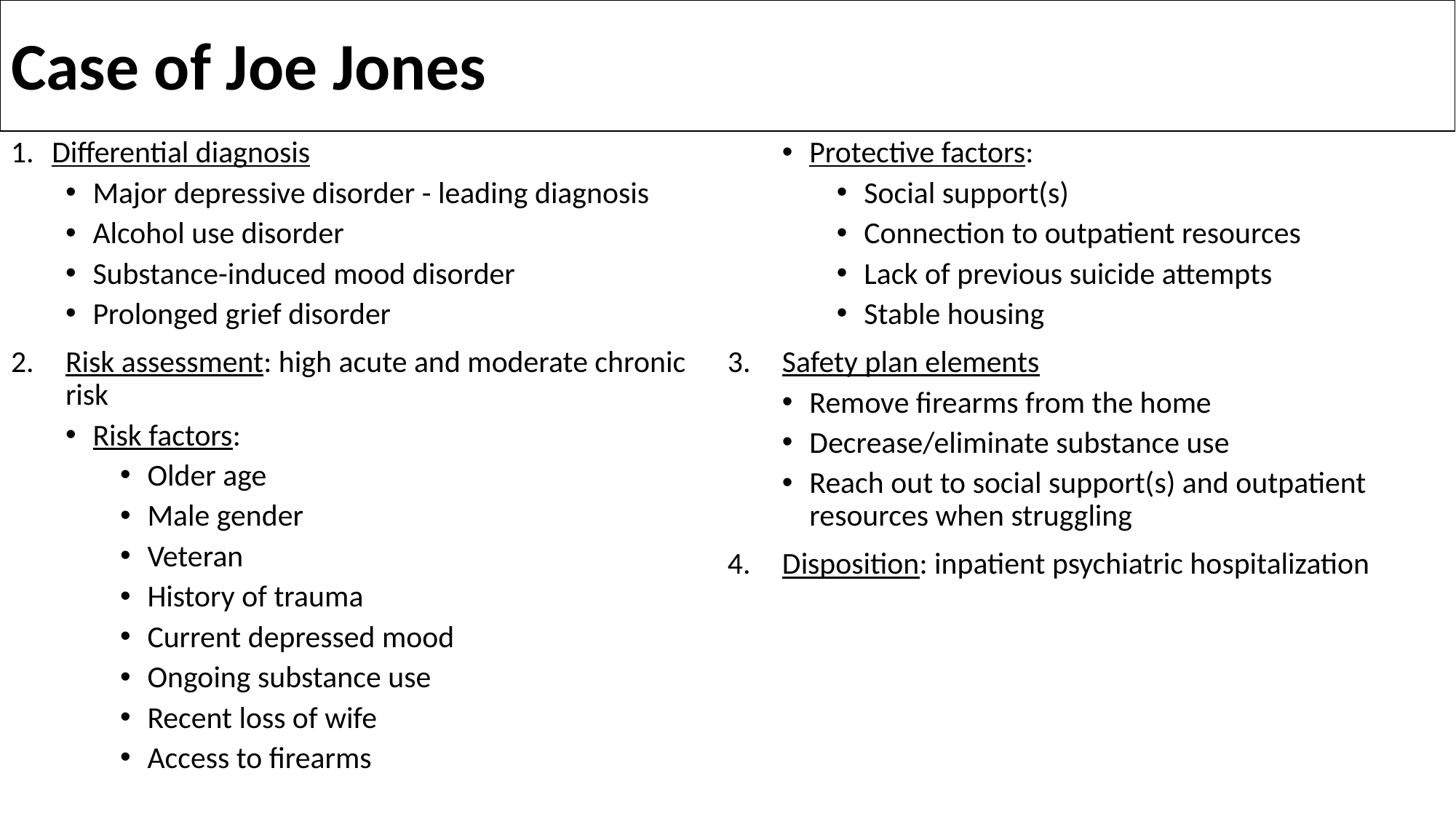

Case of Joe Jones
Differential diagnosis
Major depressive disorder - leading diagnosis
Alcohol use disorder
Substance-induced mood disorder
Prolonged grief disorder
Risk assessment: high acute and moderate chronic risk
Risk factors:
Older age
Male gender
Veteran
History of trauma
Current depressed mood
Ongoing substance use
Recent loss of wife
Access to firearms
Protective factors:
Social support(s)
Connection to outpatient resources
Lack of previous suicide attempts
Stable housing
Safety plan elements
Remove firearms from the home
Decrease/eliminate substance use
Reach out to social support(s) and outpatient resources when struggling
Disposition: inpatient psychiatric hospitalization

## Slide 10
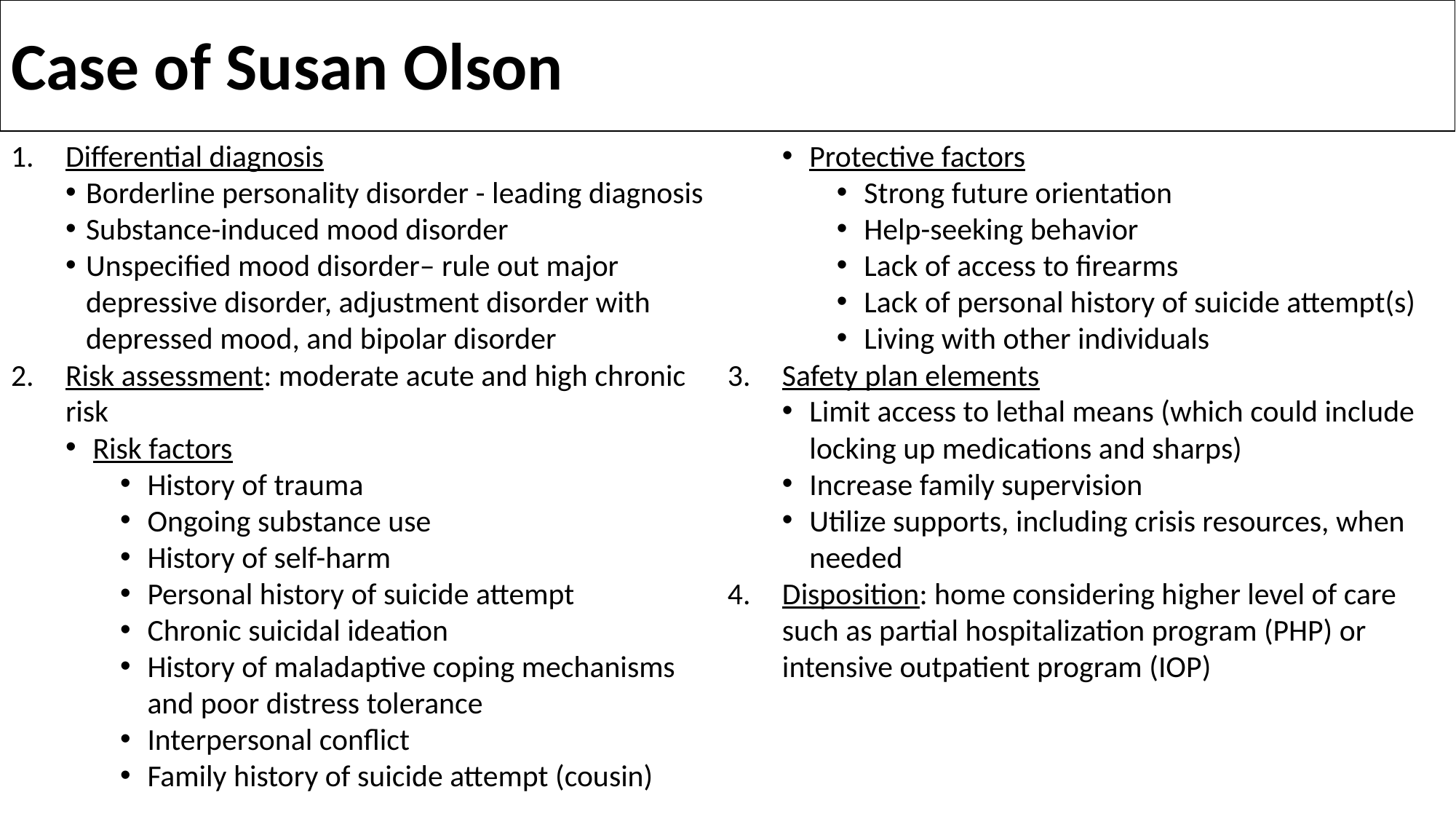

Case of Susan Olson
Differential diagnosis
Borderline personality disorder - leading diagnosis
Substance-induced mood disorder
Unspecified mood disorder– rule out major depressive disorder, adjustment disorder with depressed mood, and bipolar disorder
Risk assessment: moderate acute and high chronic risk
Risk factors
History of trauma
Ongoing substance use
History of self-harm
Personal history of suicide attempt
Chronic suicidal ideation
History of maladaptive coping mechanisms and poor distress tolerance
Interpersonal conflict
Family history of suicide attempt (cousin)
Protective factors
Strong future orientation
Help-seeking behavior
Lack of access to firearms
Lack of personal history of suicide attempt(s)
Living with other individuals
Safety plan elements
Limit access to lethal means (which could include locking up medications and sharps)
Increase family supervision
Utilize supports, including crisis resources, when needed
Disposition: home considering higher level of care such as partial hospitalization program (PHP) or intensive outpatient program (IOP)

## Slide 11
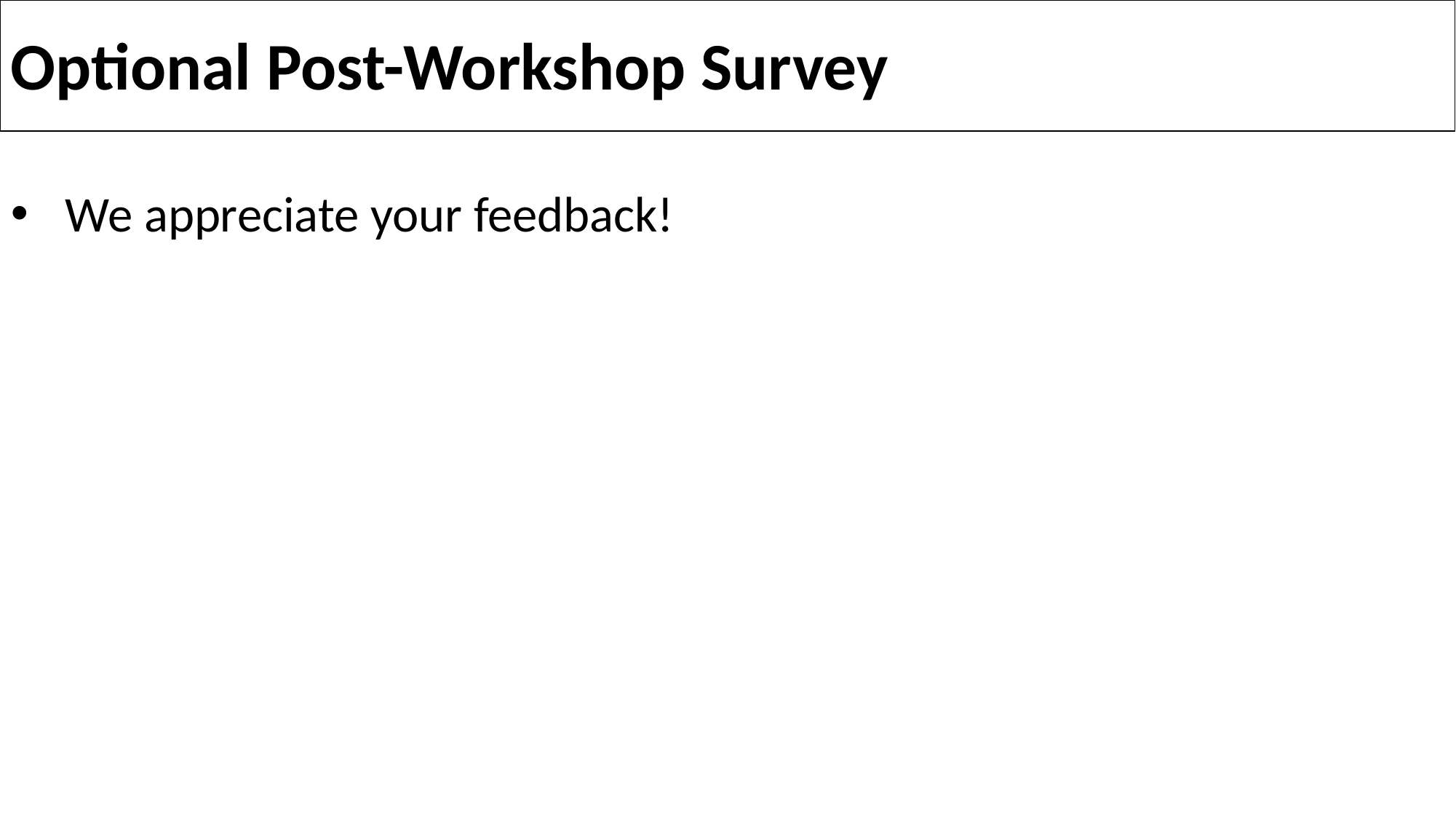

Optional Post-Workshop Survey
We appreciate your feedback!

## Slide 12
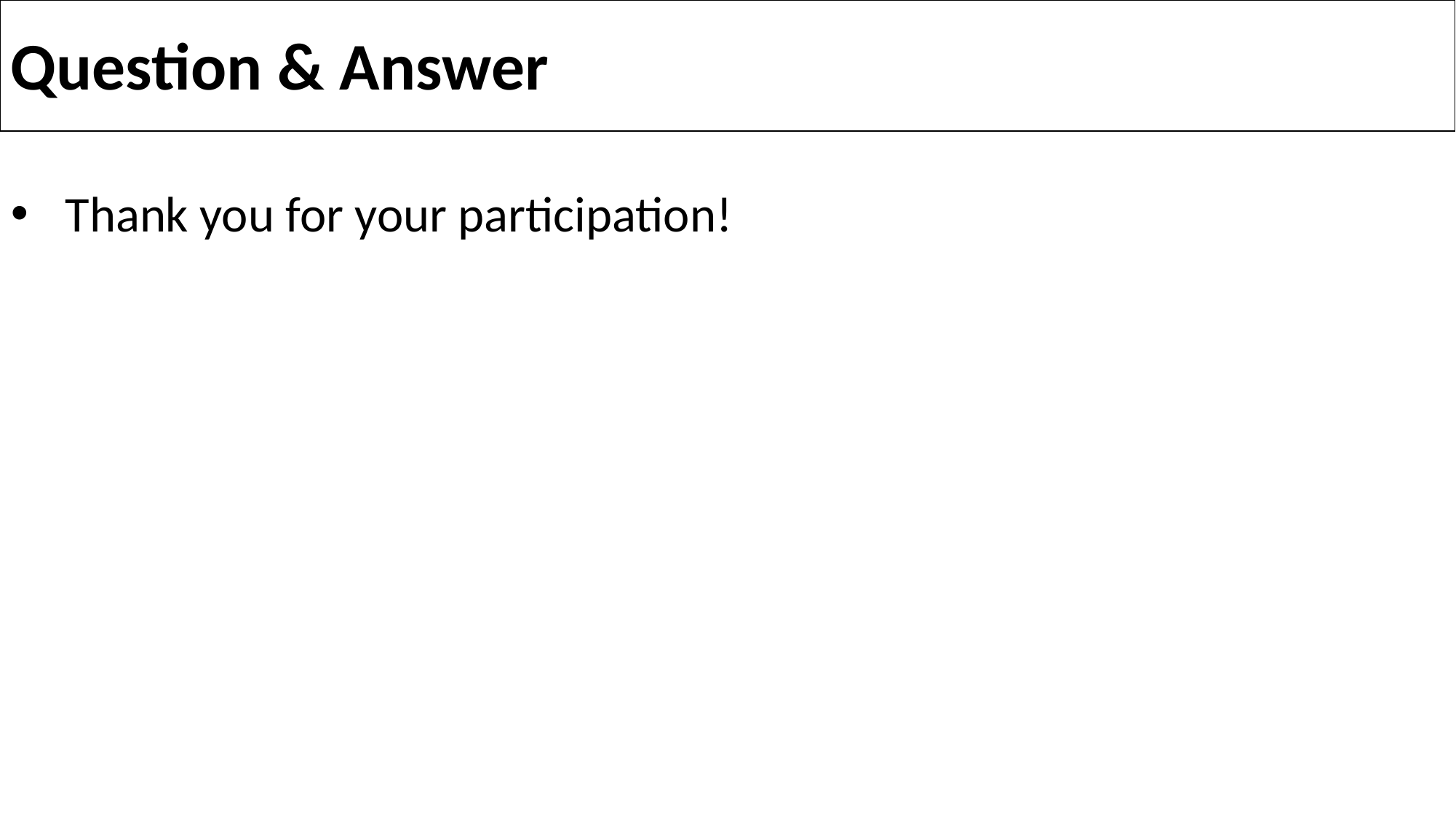

Question & Answer
Thank you for your participation!
