# Supplementary material for: A Multimodal Workshop to Improve Medical Student Self-Assessment of Knowledge and Comfort Managing Patients With Suicidality
Source: MedEdPORTAL. 2025 Jan 17;21:11488. doi: 10.15766/mep_2374-8265.11488 (PMC11739282; doi:10.15766/mep_2374-8265.11488)
Supplement: Supplementary file 1 — SP Case - Joe Jones.docxSP Case - Susan Olson.docxPreworkshop Slides.pptxDidactic and Group Discussion Slides.pptxCase of Joe Jones Door Card.docxCase of Susan Olson Door Card.docxSP encounter Facilitator Guide.docxPreworkshop Survey.docxPostworkshop Survey.docx [file mep_2374-8265.11488-s001.zip › E. Case of Joe Jones Door Card.docx]

**Appendix E. Case of Joe Jones Door Card**

*This door card should be distributed to medical students just before the standardized patient interview. This should take students approximately 1 minute to read.*

Vital signs: BP 155/90, HR 85, SpO2 100% on room air, RR 15

Patient with a history of PTSD and hypertension. Typically seen at the VA for care. Patient texted his friend today that he plans to shoot himself. Patient’s friend called police for a welfare check. Police brought the patient to the emergency department.

In triage, patient reported symptoms of depression for the last 6 months since his wife passed away. Was prescribed escitalopram/Lexapro by his primary care provider 5 months ago. He stopped taking it several months ago because “I didn’t want to take more medication and I’m not sure it was helping”.
