# Supplementary material for: A Multimodal Workshop to Improve Medical Student Self-Assessment of Knowledge and Comfort Managing Patients With Suicidality
Source: MedEdPORTAL. 2025 Jan 17;21:11488. doi: 10.15766/mep_2374-8265.11488 (PMC11739282; doi:10.15766/mep_2374-8265.11488)
Supplement: Supplementary file 1 — SP Case - Joe Jones.docxSP Case - Susan Olson.docxPreworkshop Slides.pptxDidactic and Group Discussion Slides.pptxCase of Joe Jones Door Card.docxCase of Susan Olson Door Card.docxSP encounter Facilitator Guide.docxPreworkshop Survey.docxPostworkshop Survey.docx [file mep_2374-8265.11488-s001.zip › F. Case of Susan Olson Door Card.docx]

**Appendix F. Case of Susan Olson Door Card**

*This door card should be distributed to medical students just before the standardized patient interview. This should take students approximately 1 minute to read.*

Vital signs: BP 120/75, HR 80, SpO2 100% on room air, RR 18

Patient with a history of depression and bulimia nervosa. Presents to the emergency department on her own for worsened suicidal ideation after a fight with her boyfriend. She is seeking admission to an inpatient psychiatric hospital. She drank a pint of vodka before she came to the emergency department.

In triage, she is noted to have a small, superficial laceration to one of her forearms which is no longer bleeding. She was prescribed Lexapro 10 mg daily by a psychiatrist 3 months ago. Stopped taking it and stopped following up a couple months ago because “it does not help”. Has trialed several antidepressant medications in the past but does not feel these were helpful.
